# Red-emissive carbon dot-cobalt oxyhydroxide nanosystem: A turn-on sensor for α-Glucosidase activity and inhibitor identification

**DOI:** 10.1016/j.mtbio.2025.102018

**Published:** 2025-06-23

**Authors:** Huihui Sun, Chuanyuan Gao, Yumin Yang, Changqing Liu, Han Qin, Mengyuan Tan, Jin Li, Xiaoxia Li, Kunze Du, Yanxu Chang

**Affiliations:** a State Key Laboratory of Chinese Medicine Modernization, Tianjin University of Traditional Chinese Medicine, Tianjin, 301617, China; b Tianjin Key Laboratory of Phytochemistry and Pharmaceutical Analysis, Tianjin University of Traditional Chinese Medicine, Tianjin, 301617, China; c School of Chinese Materia Medica, Tianjin University of Traditional Chinese Medicine, Tianjin, 300193, China

**Keywords:** α-Glucosidase, Förster resonance energy transfer, Inhibitors, *Polygonum cuspidatum*, R-CDs@CoOOH NCs, Type 2 diabetes

## Abstract

The development of efficient methods for sensing αlpha-glucosidase (α-Glu) and screening its inhibitors has attracted significant attention due to their pivotal role in discovering therapeutic medicines for Type 2 diabetes. Herein, a low-cost and sensitive fluorometric strategy based on red carbon dots (R-CDs) and cobalt oxyhydroxide nanosheets (CoOOH NSs) had been established to detect α-Glu and screen its inhibitory compounds in natural products. As a switched fluorescence source, the fluorescence of R-CDs at 625 nm could be quenched by CoOOH NSs via Förster resonance energy transfer (FRET), assembled into nonfluorescent R-CDs@CoOOH nanocompositecomposites (R-CDs@CoOOH NCs). α-Glu hydrolyzed L-ascorbic acid-2-*O*-*α*-D-glucopyranose to produce ascorbic acid, which could reduce CoOOH NSs to Co^2+^, destroying R-CDs@CoOOH NCs and restoring the emission of red fluorescence. The proposed method exhibited a linear α-Glu range from 0.01 to 15 U mL^−1^ and a low limit of detection (LOD) of 0.0037 U mL^−1^. Meanwhile, high-performance liquid chromatography-DAD-fraction collector (HPLC-DAD-FC) had been employed and combined with ultra-high-performance liquid chromatography-triple quadrupole time-of-flight mass spectrometry to isolate, enrich, and characterize compounds from *Polygonum cuspidatum* (*PC*). This strategy was further extended by integrating the fluorometric platform with the HPLC-DAD-FC system to explore the inhibitory effects of *PC* extracts and anti-diabetic ingredients. Finally, 85 constituents were identified, with seven compounds exhibiting high α-Glu inhibitory activity. Consequently, the established strategy could accurately determine α-Glu in vitro and screen its inhibitors from natural products.

## Highlights

• A sensor based on red carbon dots and cobalt oxyhydroxide nanosheets were prepared.
• The fluorometric platform was applied to detect α-Glu in *vitro* with simple procedure.
• The sensor as a switched signaling source was used for screening inhibitors.
• Seven antidiabetic ingredients of *Polygonum Cuspidatum* were assessed.

## Graphical abstract

**Figure:**
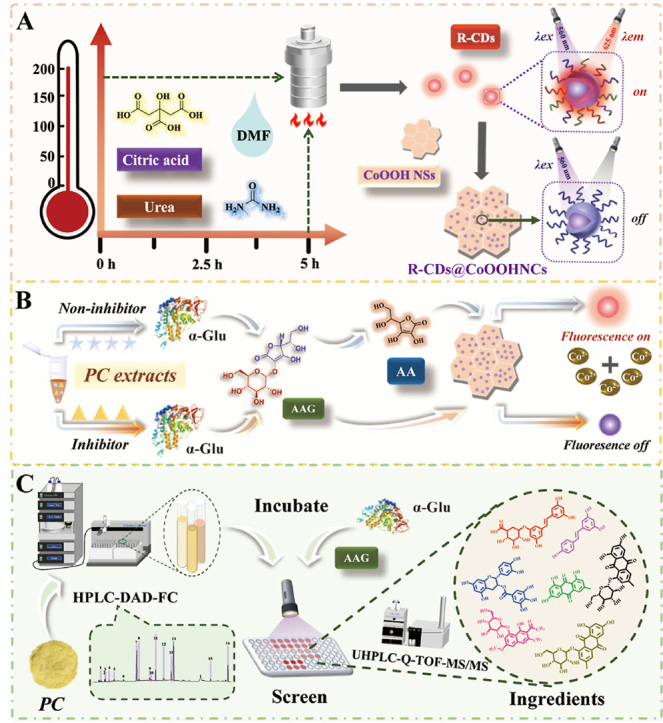

## 1 Introduction

Diabetes, a chronic metabolic disease characterized by metabolic endocrine disorders, primarily arise from insufficient insulin secretion, pancreatic islet dysfunction, and external factors, garnering extensive attention worldwide [[1], [2], [3]]. Among different types, type 2 diabetes (T2D) is recognized as one of the major forms. Persistent postprandial hyperglycemia tends to trigger multiple complications, such as diabetic nephropathy, diabetic retinopathy, stroke, foot ulcers, neuropathy, and cardiovascular diseases [4,5]. α-Glucosidase (α-Glu) is a hydrolyzing enzyme that is remarkably important in regulating glucose levels within the normal range, accelerating the release of glucose during digestion. The screening and use of α-glucosidase inhibitors have been a critical approach in the current prevention and treatment of T2D [6]. Individuals with T2D manage blood glucose levels by taking oral α-Glu inhibitors such as acarbose, miglitol, and voglibose [7]. Unfortunately, prolonged use of these medications often induces some adverse reactions, such as ileus, flatulence, nausea, and vomiting [8,9]. Therefore, there is great significance for accurate quantitative analysis and detection of α-Glu and screening for inhibitors.

The classical method for α-Glu sensing is p-nitrophenol glucopyranoside colorimetry due to its practicality and simplicity. In contrast, this method was prone to overlap absorptions, high interference, and low sensitivity. Subsequently, although some emerging approaches for assaying α-Glu and high-throughput screening were determined by using nuclear magnetic resonance (NMR), capillary electrophoresis (CE) [10], colorimetric, and electrochemical methods [11], they exhibited several inevitable shortcomings [12]. For example, the long wait required by NMR makes it difficult to improve the screening efficiency, CE is highly susceptible to environmental factors, and colorimetry has a relatively low sensitivity. In addition, electrochemistry has significant interference issues [12,13]. Noteworthily, fluorescent technology sensing has attracted much attention due to its rapidity, simple operation, and high sensitivity [[14], [15], [16]].

Carbon dots (CDs) are nanomaterials composed of carbon arranged into spherical or quasi-spherical shapes with dimensions less than 10 nm and exhibit unique optical, electronic, and chemical properties [17,18]. They have obvious merits, such as low carbon sources, superior chemical stability, high water solubility, no fear of photobleaching, good biocompatibility, and low toxicity, which enable them to become the next generation of fluorescent materials for biosensing. CDs can access the specificity and high affinity of the corresponding targets through surface modification recognition groups. Liang et al. found that *β*-cyclodextrin and blue carbon dots (CDs) can be assembled with the assistance of phenylboronic acid into a multifunctional CDs (mCDs) nanoplatform for sensitive analysis of α-Glu and screening its inhibitors. The high recognition and intense binding ability of the hydrolyzed product p-nitrophenol to *β*-cyclodextrin and phenylboronic acid effectively quenched the fluorescence of mCDs [19]. However, most CDs emit bright blue-green light under UV lamps and have relatively low quantum yields (QY) in the long wavelength range, leading to low sensitivity and a narrow detection range [20]. When screening for inhibitors, they are susceptible to interference from autofluorescence in some natural sample matrices, which can yield spurious results. Recent research findings have demonstrated that optimizing the reaction conditions and solvent polarity for synthesis resulted in red-shifting of the emission wavelength and substantially increased quantum yield [21,22]. The excellent characteristics of long-wavelength red carbon dots show great potential for applications, which will have beneficial impacts in environmental and biomedical fields. Cobalt oxyhydroxide nanosheets (CoOOH NSs) are 2D sheet nanomaterials with a large specific surface area made from abundant and low-cost raw materials [23]. They are easily synthesized, with adjustable size and good oxidizability. Due to their exceptional properties and good stability, they have attracted interest in optical analysis, electrocatalysis, and biocatalysis, and have been widely used as effective fluorescence quenching agents for developing sensing platforms. Although CoOOH NSs have been reported to have excellent quenching ability, the construction of fluorescent sensing platforms with active nanoplatforms built from R-CDs and CoOOH NSs for detecting α-Glu has barely been reported [[24], [25], [26], [27], [28]].

Screening enzyme inhibitors from natural products is a promising disease prevention and treatment approach due to their green, low-toxic, and effective properties [29,30]. *Polygonum cuspidatum* is the dried rhizome and root of *Polygonum cuspidatum Sieb. et Zucc.*, has efficacy in clearing away heat and dampness, dispelling yellowing, and dissipating blood stasis [31]. Experimental research has revealed that it can be used to treat inflammation, diabetes, gout, cancer, viral disorders, bacterial infectious diseases, and neurodegenerative diseases [32,33]. Some studies have depicted that ethyl acetate and ether extracts of *R. japonicus*, *R. megacephalus*, and *R. bohemiaChrtek* and *Chrtkova* exhibit higher anti-glycolytic oxidative activity compared with aminoguanidine [34,35]. Resveratrol well reduces glucose levels in blood, protects pancreatic B cells, and improves insulin resistance [36,37]. In addition, Polydatin ameliorates salivary insufficiency due to diabetes-induced salivary insufficiency in db/db type 2 diabetes model mice [38]. Although resveratrol-4′-*O*-D-Glucoside, polydatin, and malonyl glucoside resveratrol from *Polygonum cuspidatum* have been found to have a good inhibitory effect in previous research [38,39], the components of *PC* that are resistant to α-glucosidase activity remain incomprehensive. Utilizing the introduced with or without inhibitors screened from *PC*, this study seeks to monitor the fluorescence changes of CDs@CoOOH NCs and provides a comprehensive analysis for ingredients in inhibiting α-Glu activity.

There are other excellent methods for detecting α-Glu [[40], [41], [42]], such as metal nanoclusters and fluorescent polydopamine. This work not only applied the excellent quenching ability of CoOOH NSs to establish a fluorescence sensing system, but also used the long emission wavelength of R-CDs to avoid some self-interference when screening natural inhibitors. A sensitive nanocomposite sensor had been developed to utilize the R-CDs@CoOOH NCs in fluorometric mode to detect α-Glu and screen inhibitors from *PC* in our pursuit. Firstly, R-CDs were synthesized by citric acid and urea under the addition of N, N-dimethylformamide (DMF), which emitted intense red fluorescence at 625 nm. Then, CoOOH NSs via Förster resonance energy transfer (FRET) quenched the fluorescence of R-CDs and self-assembled to form a composite nanomaterial, R-CDs@CoOOH NCs. α-Glu hydrolyzed the substrate AAG to release AA, which effectively suppressed the quenching ability of CoOOH NSs, resulting in the restoration of fluorescence. In the system, various conditions were optimized, such as the pH of PBS, volume ratio, and time for establishing the fluorescence sensor. The amounts of R-CDs used in this work to prepare the fluorescence nanosensor showed a wider detection range and lower detection limit for detecting α-Glu compared with previous literature. Additionally, the approach was further applied to screening α-Glu inhibitors extracted from *PC* by connecting the HPLC-DAD-FC system. This proposed strategy has high specificity and sensitivity, which not only expanded the avenue for the design of biosensors and sensing strategies for monitoring α-Glu but also provided a new direction for screening active compounds in traditional Chinese medicine for treating diseases.

## 2 Materials and methods

### 2.1 Materials and apparatus

*Polygonum cuspidatum* was purchased by Beijing TongRenTang (Beijing, China). Anhydrous glucose, Chitosan (CS), Lipase, Pepsin, Tyrosinase, Pancreatin, α-amylase, bovine serum albumin (BSA), glutathione (GSH), Leu, Gly, L-Trp, α-Glucosidase, β-Glucosidase, and ascorbic acid (AA) were obtained from Shanghai Yuanye Bio-Technology Co., Ltd. Cobalt chloride, sodium hypochlorite, N, N-dimethylformamide, urea, and citric acid were purchased from Shanghai Yien Chemical Technology Co., Ltd. (Shanghai, China). Hydrochloric acid and sodium hydroxide were purchased from Sinopharm Chem. Ltd (Beijing, China). Sodium chloride (NaCl), magnesium sulfate (MgSO_4_), sodium carbonate (Na_2_CO_3_), ferrous chloride (FeCl_2_), calcium chloride (CaCl_2_), sodium bicarbonate (NaHCO_3_), disodium hydrogen phosphate (Na_2_HPO_4_), and magnesium chloride hexahydrates (MgCl_2_^.^6H_2_O) were obtained from Tianjin Chemical Reagent Co., Ltd. (Tianjin, China). Polydatin (DSTDH003802), (−)-Epigallocatechin gallate (DST191026-037), Emodin-8-*O*-D-glucoside (DST230407-066), and Emodin (DSTDD003004) were all purchased from Chengdu Desite Biotechnology Co., Ltd. Resveratrol (JF-STANDARD) was purchased from Tianjin Jianfeng Natural Products Co., Ltd. Emodin-1-*O*-*β*-D-glucoside (PS1718-0010 MG), Torachrysone-8-*O*-*β*-D-glucoside (PS1938-0010 MG), cysteine (PS020874), and homocysteine (PSD241118-011) were purchased from Chengdu Pusi Biotechnology Co., Ltd.

Chromatographic data and mass spectrometry data were measured separately using U3000 (ThermoFisher Scientific, USA) and a 1290 UHPLC system equipped with a 6546 LC/Q-TOF mass spectrometer (Agilent Corporation, Santa Clara, CA, USA). Fluorescence spectra and UV–Vis absorption spectra were measured using a multifunctional enzyme marker. Transmission electron microscopy (TEM) images and field emission transmission electron microscopy (HRTEM) images from the Tecnai VG2 F30 microscope (FEI, USA) were obtained. FTIR spectra were carried out by a Nicolet 6700 (Thermo Nicolet Corporation, USA) IR spectrometer. Zeta potential was measured by dynamic laser scattering (ZS90, Malvern). X-ray photoelectron spectra were carried out Escalab 250XI (Thermo Fisher Scientific, USA). X-ray diffraction (XRD) patterns were obtained by a Bruker D8 Advance diffractometer (ADVANCE, German). Fluorescence lifetime was measured with a photocounting spectrometer (Edinburgh Instruments FLS900, UK).

### 2.2 Synthesis of the R-CDs@CoOOH NCs

The R-CDs were synthesized by referencing the previous literature report with minor revisions [43]. Briefly, 1.0 g of anhydrous citric acid and 2.0 g of urea were weighed and dispersed in 10 mL of N, N-dimethylformamide. After fully mixing, the mixture was placed in a 50 mL polytetrafluoroethylene (PTFE) autoclave set to 180 °C for 5 h. Following cooling to room temperature, 20 mL of NaOH solution (50 mg mL^−1^) was added to the mixture and stirred for 5 min. Then, 1.25 mL of concentrated hydrochloric acid was gradually put into the above solution and agitated for 10 min. After stirring, the reaction solution was dialyzed with ultrapure water over a semi-permeable membrane for 4 h. The dialysate was centrifuged at 10000 rpm for 20 min. After dialysis purification, the precipitate was freeze-dried to obtain R-CDs. For the subsequent experiment, R-CDs were stored in a refrigerator at 4 °C.

The preparation of CoOOH NSs was conducted according to a previously worked method with some modifications [44,45]. In short, 1.5 mL of NaOH (1.0 mM) was added to 50 mL of CoCl_2_ (10 mM) solution and sonicated for 5 min, followed by centrifugation at 7000 rpm for 20 min. Then, the sediment was redispersed in ultrapure water, and 2.5 mL of NaClO solution (0.9 M) was dripped into the above mixture and sonicated for 20 min. Subsequently, the CoOOH NSs were collected by centrifuging at 7000 rpm for 10 min and purified with water three times. Finally, the precipitated CoOOH NSs were prediluted with deionized water to 50 mL for further experiments.

The R-CDs@CoOOH NCs should be prepared for mixing with 40 μg mL^−1^ R-CDs aqueous solution, CoOOH NSs aqueous solution, and ultrapure water thoroughly in volume: 5:2:8. The mixed solution was stood for 20 min at 37 °C before further experimentation.

### 2.3 Detection of ascorbic acid

First, 150 μL R-CDs@CoOOH NCs solution was mixed with 250 μL pH 6.8 PBS buffer solution, and ultrapure water was added to a total of 500 μL, followed by the addition of AA with various concentrations. The mixed solution was incubated at 37 °C for 40 min. Ultimately, the signals of fluorescence were recorded in the fluorescence spectra under an emission wavelength of 625 nm.

### 2.4 Fluorometric assay of α-Glu

Various concentrations of 100 μL α-Glu were mixed with 100 μL of AAG (10 mM) in 0.1 M PBS buffer (pH 6.8) for 30 min. Then, 150 μL of R-CDs@CoOOH NCs were added, and the solution was incubated at 37 °C for 40 min. Therefore, the fluorescence spectra were measured.

### 2.5 Sample preparation

One gram of *PC* powder was immersed in 50 % methanol solution, sonicated for 30 min, and centrifuged. The supernatant was concentrated, dried, and completely dissolved in 50 % methanol, and the precipitate was discarded by centrifugation at 13400 rpm for 6 min. The filtrate obtained after filtration through a 0.22 μM pore size filter was then pumped at high pressure into the HPLC run for 60 min to separate. In order to achieve the desired separation and collection status, we optimized the ratio of mobile phases and the separation time. And the HPLC was connected to an automatic fraction collector to collect the fractions according to the time mode. The fractions collected after 4 enrichments were dried with nitrogen and redissolved in methanol to obtain 16 sample fractions.

### 2.6 Chromatographic conditions of fraction collection

The chromatograms of extracts of *PC* were performed by an HPLC system combined with photodiode array detection (DAD) at an injection volume of 10 μL and a column temperature of 25 °C. An Agilent HPLC Eclipse plus C18 (5 μm, 4.6 × 250 mm) was used for analysis. The mobile phase of gradient elution was 0.1 % formic acid (A) and acetonitrile (B). It has proceeded with 7–17 % of B in 0–6 min; 17 % of B in 6–14 min; 17–20 % of B in 14–15 min; 20–25 % of B in 15–25 min; 25–28 % of B in 25–28 min; 28–30 % of B in 28–30 min; 30 % of B in 30–42.5 min; 30–40 % of B in 42.5–43 min; 40–45 % of B in 43–50 min; 45–90 % of B in 50–60 min with a flow rate of 1 mL min^−1^.

### 2.7 α-Glu inhibitors screening

Firstly, the solution involved mixing 100 μL of α-Glu (10 U mL^−1^) with 140 μL of PBS (0.1 M, pH 6.8), and 10 μL of acarbose and antidiabetic active ingredients from *PC* with various concentrations were added, and then incubated at 37 °C for 20 min. Secondly, 100 μL of AAG (10 mM) was added and incubated at 37 °C for 30 min. Finally, 150 μL of R-CDs@CoOOH NCs were followed by another 40 min before recording the fluorescence emission spectrum. The inhibitor rate (%) formula was as follows: Inhibitor (%) = 1-(F_2_-F_1_)/(F-F_0_), where F_0_ was the fluorescence intensity of R-CDs@CoOOH NCs without α-Glu, F was the fluorescence intensity of R-CDs@CoOOH NCs in the presence of α-Glu, F_1_ was the fluorescence intensity of R-CDs@CoOOH NCs with inhibitors at the same concentration, and F_2_ was the fluorescence intensity of R-CDs@CoOOH NCs in the presence of α-Glu and inhibitors.

### 2.8 Characterization of PC extracts

The *PC* extracts were easily analyzed qualitatively on the 1290 UPLC system equipped with a 6546 LC/Q-TOF mass spectrometer (Agilent Corporation, Santa Clara, CA, USA) with an electrospray ionization (ESI) source. The ratio of the mobile phase and the separation time was simply optimized. The column for analysis was an Agilent UPLC Eclipse XDB-C18 (1.8 μm, 2.1 × 150 mm) with a guarded column of Agilent Eclipse XDB-C18 (1.8 μm, 2.1 × 5 mm). The mobile phase of gradient elution was 0.1 % formic acid (A) and acetonitrile (B). It has proceeded with 5–50 % of B in 0–20 min; 50–92 % of B in 20–30 min with a flow rate of 0.3 mL min^−1^. A series of analytical procedures was completed with an injection volume of 3 μL and a column temperature of 30 °C based on the default supporting information acquisition settings. In order to provide comprehensive information for the structural identification of the sample, both negative and positive ion modes were registered. The collision energy in MS/MS analysis was set at 10 V and 30 V, and the mass range (*m/z*) was set as 50 to 1000. The obtained ion fragments of MS and MS^2^ were summarized on SCIEX OS software to find out the molecular formula, relative molecular mass error, and characteristic ion fragments of various compounds of *PC*. The results were compared to published literature, PubMed, PubChem, and ChemSpider. The chemical structure information was used to determine the chemical constituents in the extracts of *PC.*

### 2.9 Molecular docking

The molecular structures of acarbose and active compounds were obtained from the PubChem database (https://pubchem.ncbi.nlm.nih.gov), processed by Auto Dock Tools, and exported as pdbqt format files. The crystal structure of α-Glu (PDB no. P3A4A) was collected from the Protein Data Bank for molecular docking into the binding site pocket. Auto Dock Vina software [46] was used as the docking engine to present the binding energy. Gasteiger charge [47] was employed to calculate electrostatic interaction. Target protein and ligands were imported together into the Pymol software (www.schrodinger.com/) to dehydrate, hydrogenate, and charge distribution. Ultimately, the dominant conformation with the lowest binding energy was selected to visualize the 3D structure.

## 3 Results and discussion

### 3.1 Synthesis and characterization of R-CDs@CoOOH NCs

**Fig. 1:**
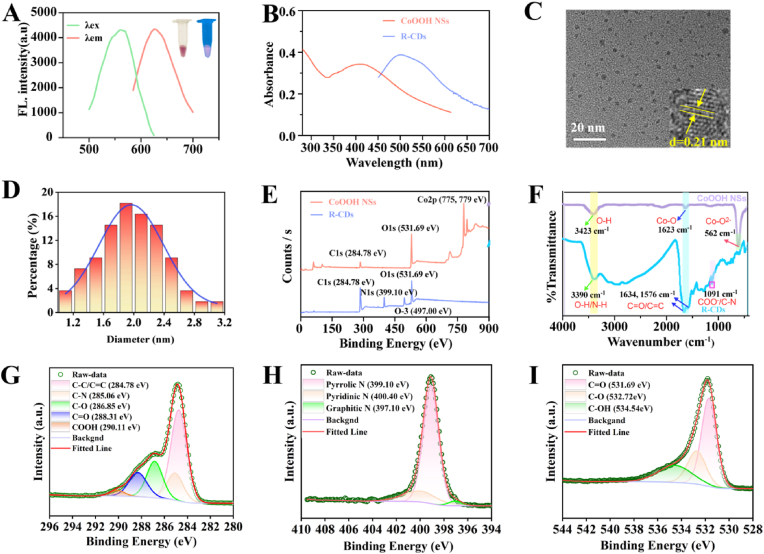
(A) Fluorescence emission spectra and (B) UV–Vis absorption spectra of R-CDs. HRTEM (C) and the size distribution (D) of R-CDs. (E) XPS of R-CDs and CoOOH NSs. (F) FTIR of R-CDs and CoOOH NSs. XPS patterns of C1s (G), N1s (H), and O1s (I) of R-CDs.

Citric acid and urea have no fluorescent emission signals as the precursors of R-CDs, while they emit red fluorescence under UV light when heat-treated at 180 °C and subsequently purified to yield a purple solution, indicating the generation of R-CDs in Fig. 1A. In addition, it could be seen that compared to unpurified CDs and purified CDs in Fig. S1, the excitation and emission of the fluorescence spectra of purified CDs were unitary, demonstrating the uniformity in the optical properties of R-CDs [48]. The fluorescence intensity was measured over seven days, and the fluorescence value remained nearly unchanged, indicating the stability of the R-CDs (Fig. S2). The fluorescence excitation wavelength was finally determined to be 560 nm. The max emission peak appeared at 625 nm in Fig. 1A. R-CDs exhibited a broad UV–Vis absorption band in the range of 450–550 nm, with the maximum absorption peak at 500 nm (Fig. 1B). The high-resolution TEM image showed the circular shape of the R-CD, with a visible crystalline lattice spacing of 0.21 nm (Fig. 1C), which was consistent with the previous report [49]. The TEM image of R-CDs indicated they had good monodispersed with diameters ranging from 1 to 3.2 nm, and the highest percentage of carbon point particles with a particle size of about 2.0 nm in Fig. 1D. Then, the elemental compositions of R-CDs we investigated with an X-ray spectrometer. The full XPS spectrum showed three strong signals at 284.78, 399.1, and 531.69 eV, verifying that the R-CDs mainly consisted of C, N, and O elements (Fig. 1E). Fourier transform infrared spectroscopy (FTIR) spectra analysis (Fig. 1F) showed that the R-CDs samples displayed a characteristic band at 3400 cm^−1^, which was considered as the stretching vibration of O-H/N-H. Additional characteristic peaks at 1634 cm^−1^ and 1576 cm^−1^ represented the stretching vibrations of C=O and C=C, respectively. And the stretching vibrations of C-N and COO- were shown at 1091 cm^−1^. As shown in Fig. 1G-I, the C1s peaks were deconvoluted at 284.78, 285.06, 286.85, 288.31, and 290.11 eV into five peaks attributed to C-C/C=C, C-N, C-O, C=O, and COOH bonds, respectively. The three deconvolution peaks generated in response to the N1s peaks were noted at 397.06, 399.09, and 400.02 eV for pyridine C-N-C, pyrrole C-N-C, and graphitic C-N=C motifs, respectively. The high-resolution O1s peaks included three peaks at 531.69, 532.72, and 534.54 eV, respectively, assigned to C=O, C-O, and C-OH bonds. The XRD pattern showed three typical peaks at 27.35°, 31.68°, 45.42°, and 56.43°, as shown in Fig. S3, which met the criteria on the JCPDS card (PDF number: 01-074-2138) and further indicate the successful synthesis of crystalline R-CDs. These results implied that the formation of pyrrole- and pyridine-conjugated cores contributes to enhancing the long-wavelength fluorescence emission properties of carbon dots. Combined with the XPS, FTIR, and XRD data, the results showed that the surface of R-CDs was enriched with abundant hydrophilic groups such as amino and carboxyl groups, ensuring their excellent solubility in water.

**Fig. 2:**
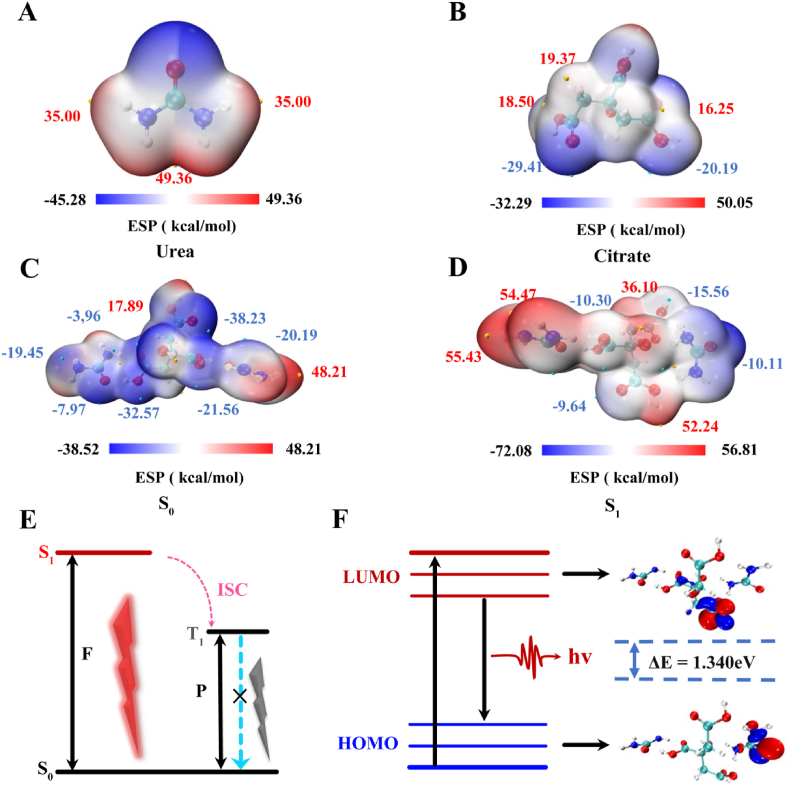
The ESP distribution map of (A) Urea and (B) Citrate. The (C) S_0_ and (D) S_1_ conformation of R-CDs. (E) Mechanism diagram of fluorescence emission. (F) LUMO and HOMO conformation of R-CDs. ISC: intersystem crossing.

In addition, molecular simulations of the red carbon dots synthesized from citric acid and urea was performed using density functional theory (DFT) [50,51] with Gaussian software. First, the ESP distribution maps were utilized to analyze the formation process of red carbon dots. Fig. 2A and B illustrated the most hydrogen-bonding-prone regions of the ESP distribution maps for citric acid and urea and quantified the ESP on the surface of both feedstocks. The local maxima and minima of the ESP were represented by red and blue colors, respectively. The blue regions were negatively charged and nucleophilic to act as hydrogen bonding donors, which endowed them with the ability to attack electrophilic atoms. In contrast, the red area was electrophilic to act as a hydrogen bonding acceptor [52,53]. For example, the elemental N in urea was distributed in the red area with an observable ESP local maximum of 49.36 kcal mol^−1^, which could be treated as a hydrogen bond acceptor. As a hydrogen bond donor, the hydrogen atoms of the carboxyl group on citric acid were distributed in the blue area, which had an ESP maximum of −32.29 kcal mol^−1^. The results of the molecular simulations showed that hydrogen bond addition was shown during the synthesis of R-CDs. Then we simulated the molecular states of the red carbon dots in the ground and the first excited states (Fig. 2C and D). To further explore the luminescent properties, the electron cloud changes of the carbon dots and the energy transfer in the HOMO and LUMO molecular orbitals (ΔE = 1.340 eV) was calculated as shown in Fig. 2E and F. It confirmed that R-CDs were successfully synthesized and possessed photoluminescent properties.

**Fig. 3:**
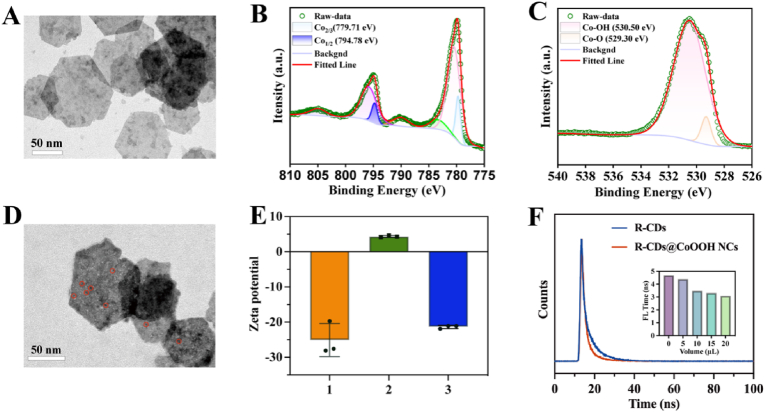
(A) TEM image of the CoOOH NSs. XPS spectra of Co2p (B) and O1s (C) of CoOOH NSs. (D) TEM image of the R-CDs@CoOOH NCs. (E) Zeta potential of (1) R-CDs, (2) CoOOH NSs, and (3) R-CDs@CoOOH NCs. (F) Fluorescence lifetime image of R-CDs (50 μL) and R-CDs@CoOOH NCs (20 μL). Inset: fluorescence lifetime of CoOOH NSs added in various volumes. Error bars are the standard error of the mean.

The CoOOH nanosheets (CoOOH NSs) displayed a typical two-dimensional sheet compound with several folds and ruffles with an average diameter of about 84 nm (Fig. 3A). CoOOH NSs exhibited a characteristic absorption peak at 410 nm (Fig. 1B). The strong signals at 285, 532, 775, and 779 eV in the XPS analysis plots of the CoOOH NSs indicated the presence of the elements C, O, and Co in the as-prepared nanosheets (Fig. 1E). The peaks at 775.0 and 779.9 eV were respectively attributed to Co2p1/2 and Co2p2/3. The O1s plot (Fig. 3B and C) of Co-OH and Co-O bonds responded to two peaks at 530.4 and 529.3 eV, respectively. As shown in Fig. 1F, the characteristic peaks in the FTIR spectra centered at 3423, 1623, and 562 cm^−1^ come from O-H, Co-O, and Co-O^2-^ bond absorption, respectively. These data verified the successful preparation of CoOOH NSs. The XRD pattern in Fig. S4 showed three typical peaks of 23.46°, 45.46°, and 59.45°, which met the criteria on the JCPDS card (PDF number: 07–0169), further indicating the successful synthesis of CoOOH NSs.

R-CDs@CoOOH NCs were easily obtained by mixing and incubating R-CDs for 20 min. In the TEM image of R-CDs@CoOOH NCs, it was revealed that R-CDs were well attached to the surface of CoOOH NSs (Fig. 3D). The loading rate of R-CDs could reach up to 87.33 % (Fig. S5). The detailed calculation method was presented in the Supplementary Materials. The charge distribution showed that R-CDs were negatively charged, CoOOH NSs were positively enriched, and the mixed system of R-CDs@CoOOH NCs was negatively charged. As shown in Fig. 3E, the specific Zeta potentials were −25.13, 4.36, and −21.37 eV, respectively. The results of TEM and Zeta potentials indicated that the R-CDs were attached to the surface of CoOOH NSs by electrostatic interactions. Furthermore, the characterization results deduced that the complexation of hydroxyl groups on R-CDs with CoOOH NSs, as evidenced by characterization data, likely mediates their binding interaction [45]. As shown in Fig. 1A and B, the emission spectra of R-CDs and the UV absorption spectra of CoOOH NSs were the prerequisites for the quenching mechanism. We also examined the fluorescence lifetimes of R-CDs before and after the addition of CoOOH NSs in Fig. 3F. The fluorescence lifetimes of R-CDs with CoOOH NSs were changed from 4.66 to 3.02 ns after incubation, elucidating the fluorescence quenching mechanism as FRET [7]. In addition, the fluorescence lifetime plots of R-CDs supplemented with various volumes of CoOOH NSs were also shown in the inset of Fig. 3F. The findings indicated that the fluorescence of the system decreased as the amount of CoOOH NSs increased with the same quantity of R-CDs.

### 3.2 Detection mechanism and feasibility study

CoOOH NSs were added to the R-CDs solution to quench the fluorescence emission of R-CDs at 625 nm via FRET, while it was restored upon the addition of AA (Fig. S6A). This was due to the strong capacity of AA to reduce CoOOH NCs to Co^2+^ [54]. As a substrate for α-Glu, AAG could be hydrolyzed by the enzyme to produce AA, which destroyed the structural system of R-CDs@CoOOH NCs. In Fig. S6B, we discovered AA, AAG, and α-Glu didn't interfere with the fluorescence emission of the R-CDs, but the fluorescence intensity of R-CDs@CoOOH NCs was reduced in the absence of AA or AAG and α-Glu. This result provided a favorable prerequisite for the further detection of α-Glu. Based on the ability of AA to restore the fluorescence of R-CDs, a fluorescence detection strategy for the detection of α-Glu was proposed. The amount of AA affected the chemical structure of CoOOH NSs, resulting in changes in fluorescence intensity. In Fig. S6C and Fig. S6D, the linear equation was obtained as Y = 0.0480 C_AA_ + 0.9829 (*R*^2^ = 0.9982) with the concentration of AA ranging from 0 to 12.5 μM.

### 3.3 Optimization conditions of fluorescent sensor

In order to determine the optimal conditions for fluorescence sensing, the reaction efficiencies of R-CDs, AA, and CoOOH NSs were investigated at various ratios and times, as well as the optimal pH for adapting to α-Glu. As shown in Fig. S7A, under the condition of reacting with the same volume of R-CDs, the efficiency of fluorescence quenching varied with the volume of CoOOH NSs solution. Then, 20 μL of CoOOH NSs solution markedly eliminated 50 μL of R-CDs (40 ng mL^−1^) and had no significant difference from 25 μL of CoOOH NSs, which was attributed to the excellent photobleaching ability, strong quenching ability, and large specific surface area of CoOOH NSs. In Fig. S7B, when R-CDs met CoOOH NSs, there was a considerable attenuation of the fluorescence, and the fluorescence intensity decreased with reaction time, while the fluorescence values of R-CDs@CoOOH NCs were almost invariant after 20 min of reaction. Therefore, the optimal reaction conditions of R-CDs@CoOOH NCs were determined to be R-CDs: CoOOH NSs (v/v; 5:2), and a reaction time of 20 min. According to Fig. S7C, the fluorescence recovery reached equilibrium when the reaction time of AA and R-CDs@CoOOH NCs was extended to 40 min. Thus, 40 min was deemed the optimal reaction time. And Fig. S7D indicated that α-Glu was more appropriate to function under weakly acidic conditions, and the optimal reaction efficiency was attained at pH = 6.8 of PBS. The alkaline environment inhibited the activity of α-Glu.

### 3.4 Fluorescent quantitative detection for α-Glu

**Fig. 4:**
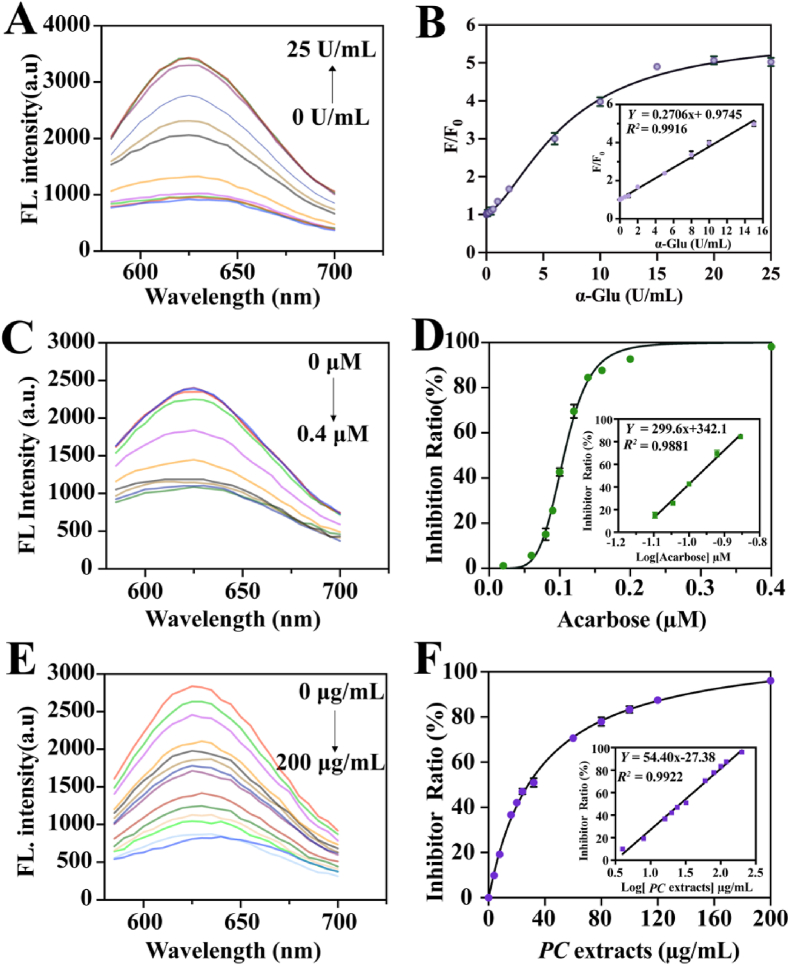
(A) The fluorescence emission spectra of R-CDs@CoOOH NCs with different concentrations of α-Glu (0–25 U mL^−1^). (B) The detection curve and linear relationship (0.01–15 U mL^−1^) of α-Glu in fluorometric mode. (C) The fluorescence emission spectra of R-CDs@CoOOH NCs with α-Glu and acarbose. (D) Inhibition ratio curve and linear relationship of acarbose in fluorometric mode. (E) The fluorescence emission spectra of R-CDs@CoOOH NCs with α-Glu and *PC* extracts. (F) Inhibition ratio curve and linear relationship of *PC* extracts in fluorometric mode.

Under the optimal conditions, the “on-off” fluorometric mode was utilized to measure α-Glu. The fluorescence intensity in the system would enhance as the amount of AA, which α-Glu catalysis AAG to produce. It could be seen that the fluorescence intensity at 625 nm was gradually improved with the increasing α-Glu concentration in the range from 0 to 25 U mL^−1^ in Fig. 4A and B. The linear equation Y = 0.2706 C_α-Glu_ + 0.9745 (*R*^2^ = 0.9926) with a range of 0.01–15 U mL^−1^ and the LOD of α-Glu was 0.0037 U mL^−1^ (based on 3σ/k rule. σ: The standard deviation of the fluorescence intensity of the blank sample; k: The slope of the calibration curve represents the linear relationship between fluorescence intensity and the concentration of α-Glu). Compared with previously reported assays, our strategy had a wider detection range and a lower detection limit in Table S1. When α-Glu activity approached 10 U mL^−1^, the fluorescence intensity recovery effect had already reached an excellent state. Therefore, 10 U mL^−1^ was chosen to screen the active ingredients for the subsequent experiments.

In addition to sensitivity, the specialty and anti-interference ability was further assessed to measure whether the sensing system was excellent. The fluorescence intensity of the system was normalized in the presence of possible interfering elements and structurally similar enzymes (Fig. S8), where F_0_ was the fluorescence intensity of R-CDs@CoOOH NCs with PBS and AAG, F was the fluorescence intensity of R-CDs@CoOOH NCs in the presence of α-Glu and AAG, and F_1_ was the fluorescence intensity of R-CDs@CoOOH NCs with interference materials and AAG. The fluorescent ratio showed that only α-Glu could significantly restore the fluorescent intensity of the system, demonstrating that it was highly selective for detecting α-Glu.

### 3.5 Inhibitor effect of acarbose and PC extracts

To estimate how the proposed method could be extended to screen α-Glu inhibitors, acarbose, a well-reported α-Glu inhibitor and one of the common oral medicines for the treatment of T2D, was selected as a model inhibitor [55]. Fig. 4C showed the fluorescence spectra of R-CDs@CoOOH NCs after the addition of different concentrations of acarbose in the range of 0–0.4 μM. The inhibitory effect of acarbose on α-Glu was concentration-dependent (Fig. 4D). IC_50_, the half-maximal inhibition of enzyme activity, was counted to be 0.1062 μM, indicating that the sensing platform could be successfully applied to screen α-Glu inhibitors. Then, this method was used to measure the inhibitory effect of *PC* extracts on α-Glu. As shown in Fig. 4E and F, increasing the concentration of *PC* extracts elevated the inhibitory effect on α-Glu, and the amount of AA produced in the system decreased, ultimately leading to a slowdown or termination of the fluorescence recovery process. The highest inhibition effect with the IC_50_ value of *PC* extracts was 36.71 μg mL^−1^. In addition, to eliminate the interference of certain reducing substances in the *PC* extracts on the fluorescence system, this work monitored the effect of different concentrations of *PC* extracts alone on the fluorescence intensity (Fig. S9A). The addition of the *PC* extracts didn't cause a significant difference in the fluorescence intensity of the detection system compared to the blank group, indicating that *PC* extracts had almost no reductive effect on CoOOH NSs. The IC_50_ value of the *PC* extracts was calculated by the traditional colorimetric method for benchmark comparison (Fig. S9B). The IC_50_ value obtained by the traditional method was 40.63 μg mL^−1^. The result was approximately closed to that of the detection method in this study, which could be the effective process for screening inhibitors under the same conditions.

### 3.6 Screening and identification of 85 antidiabetic active ingredients from PC

Based on the mixed nature and complexity of TCM extracts, HPLC was used to achieve separation. The incorporation of a fraction collector greatly improves the collection efficiency and accuracy. Because of the antidiabetic activity of *PC*, UHPLC-Q-TOF-MS/MS was applied to the positive and negative ion modes to identify the potential hypoglycemic active components of *PC.* Then 16 fractions were collected after injecting *PC* extracts into HPLC-DAD-FC for 60 min. Each fraction was tested the antidiabetic activity, and the highly active fractions were selected for identification by UHPLC-Q-TOF-MS/MS. The systems of UHPLC-Q-TOF-MS/MS separated the active components from *PC* for 30 min and were analyzed to obtain structural ion information. The results extracted from the positive and negative ion chromatograms showed that 85 compounds were identified, including 81 compounds in the negative ion (Fig. S10) and 4 compounds in the positive ion (Fig. S11). The identification process of the compounds was mainly derived from two sources. On the one hand, the ion fragmentation comparison and molecular cleavage pattern derivation from previous literature. On the other hand, the information obtained from UHPLC-Q-TOF-MS/MS. Table S2 listed the number of peaks, retention time, compound name, ionic pattern, molecular formula, mass error, and primary and secondary fragmentation ions in formation.

### 3.7 Comparison of antidiabetic activity

**Fig. 5:**
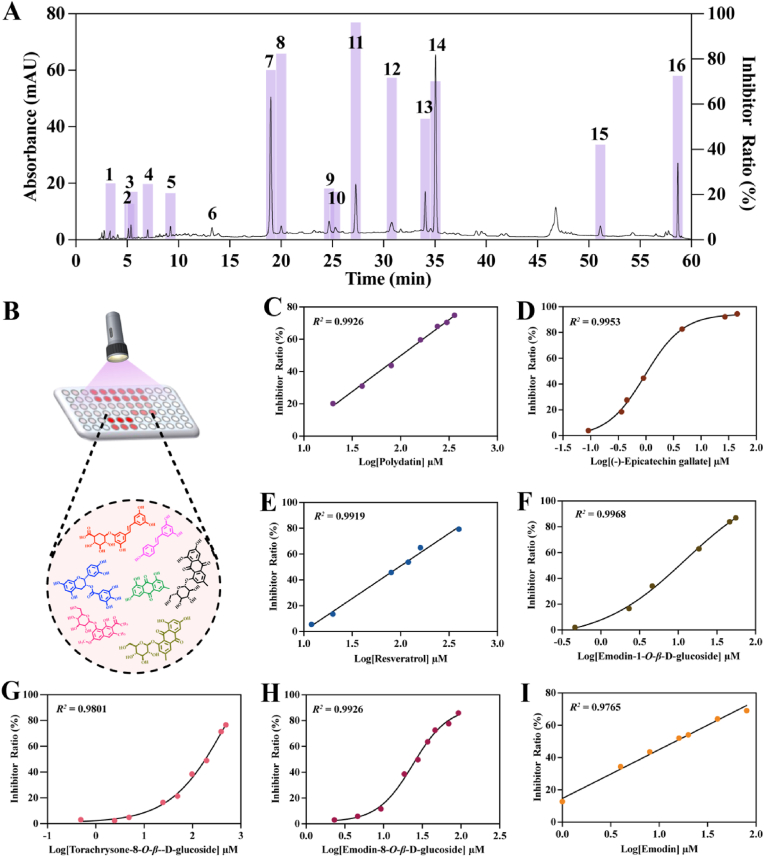
(A) The chromatogram of *PC* extracts and inhibitory rate of α-Glu activity with different fractions. The black line was the typical chromatogram of *PC*, and the bar graph was the inhibitor ratio of 16 fractions. (B) Schematic diagram of the inhibitory activity of the screened active compounds. Inhibition curves of α-Glu with seven active ingredients, including (C) Polydatin, (D) (−)-Epicatechin gallate, (E) Resveratrol, (F) Emodin-1-*O*-*β*-D-glucoside, (G) Torachrysone-8-*O*-*β*-D-glucoside, (H) Emodin-8-*O*-*β*-D-glucoside, and (I) Emodin.

Fractions were collected based on peak-out time by combining HPLC and a fraction collector. Then, the collected fractions were incubated with α-Glu separately, and the inhibition contribution of the 16 fractions was calculated. Based on the analytical results as shown in Fig. 5A, a total of seven active fractions with greater than 50 % inhibition were screened out by combining easy availability and the magnitude of inhibition. The final active ingredients were identified, including (7) polydatin, (8) (−)-epicatechin gallate, (11) emodin-1-*O*-*β*-D-glucoside, (13) resveratrol, (14) torachrysone-8-*O*-*β*-D-glucoside, (15) emodin-8-*O*-*β*-D-glucoside, and (16) emodin. The chromatograms and chemical structures of seven compounds are shown in Fig. S12 and Fig. S13. The fluorescence intensity was reduced with the increasing dose of the seven plant extracts, indicating that all seven components had a certain inhibitory effect on α-Glu. As shown in Fig. 5C-I and Table 1, (−)-epicatechin gallate showed the highest inhibition effect with IC_50_ of 0.8725 μM, followed by emodin-1-*O*-*β*-D-glucoside, emodin, emodin-8-*O*-*β*-D-glucoside, resveratrol, polydatin, and with IC_50_ of 9.953, 15.25, 27.00, 100.7, 105.3, and 215.3 μM, respectively, and the IC_50_ of torachrysone-8-*O*-*β*-D-glucoside was 215.3 μM. The results demonstrate the reliability of this fluorescent sensor.

**Table 1:** Results of molecular docking and IC_50_ values for acarbose and seven compounds with α-Glu.

Compound	Binding energy	Hydrogen bonds	Amino acid	IC_50_ (μM)
Acarbose	−8.2	7	HIS-280(2.9, 2.4, 2.1), SER-304(2.4), ASP-307(2.1), GLN-353(2.3), TYR-158(2.0)	0.1062 ± 0.0014
Polydatin	−9.3	5	TRP-468(2.3), LYS-406(2.2), TYR-407(2.1), ARG-413(2.1), GLU-408(1.8)	105.3 ± 4.7
(−)-Epicatechin-gallate	−9.9	5	TYR-158 (2.3), SER-157(3.5), ARG-442(2.7), GLU-411(2.4), SER-311(2.5)	0.8725 ± 0.1960
Emodin-1-*O*-*β*-D-glucoside	−9.5	4	ARG-315(2.4), LYS-156(2.1), TYR-158(2.9), ARG442(2.6)	9.953 ± 0.7320
Resveratrol	−8.5	5	ARG-315(2.5), GLN-353(2.0), HIS-112(2.4), GLN-182(2.7), ASP-69(1.9)	100.7 ± 5.6
Torachrysone-8-*O*-*β*-D-glucoside	−9.2	7	LEU-313(2.3), ARG-315(1.9), ARG-442(2.6), GLU-411(3.0), TYR-158(2.5), LYS-156(2.7,2.6)	215.3 ± 87.0
Emodin-8-*O*-*β*-D-glucoside	−9.8	6	PRO-312(2.4), GLN-279(2.9,2.5), TYR-158(3.4), GLU-411(2.6), ARG-315(1.9)	27.00 ± 1.25
Emodin	−8.3	5	GLN-279(2.1, 2.4), ARG-442(2.4), GLU-411(3.0), TYR-158(3.2)	15.25 ± 2.25

### 3.8 Molecular docking

**Fig. 6:**
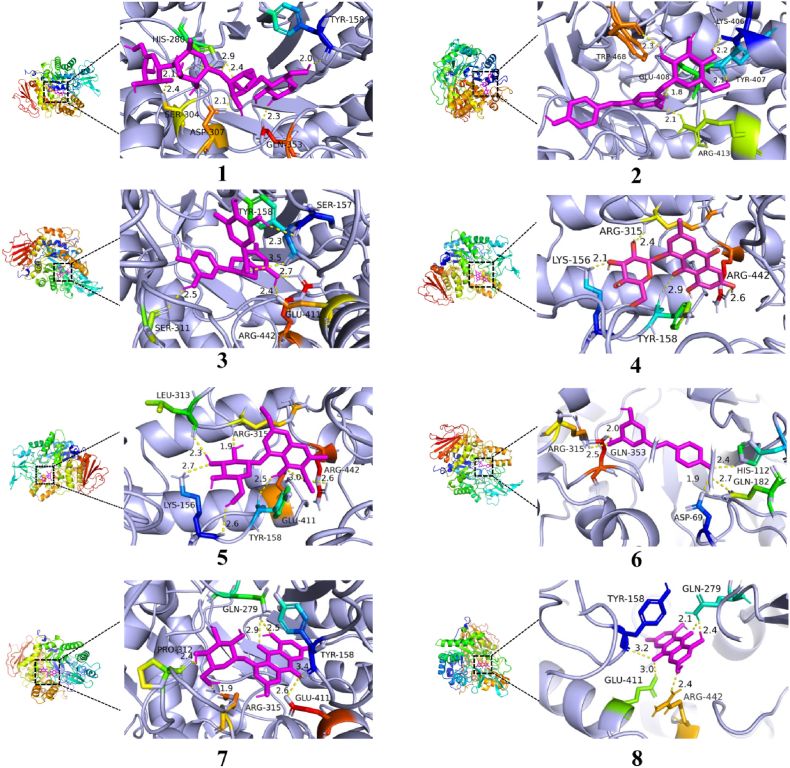
Detailed molecular docking diagrams of acarbose and the seven active ingredients with α-Glu. (1) Acarbose; (2) Polydatin; (3) (−)-Epicatechin gallate; (4) Emodin-1-*O*-*β*-D-glucoside; (5) Torachrysone-8-*O*-*β*-D-glucoside; (6) Resveratrol; (7) Emodin-8-*O*-*β*-D-glucoside; (8) Emodin.

As a typical T2D inhibitor, acarbose could markedly decrease α-Glu activity, which could balance blood glucose in *vivo*. To find out the binding between these candidate compounds and α-Glu, the binding energy and sites of acarbose and ingredients with α-Glu protein were compared via molecular docking. As shown in Fig. 6, the binding sites of seven ingredients were not only similar to those of acarbose, but also shared the same binding amino acids. The binding energies between all compounds and α-Glu protein were calculated, which meant the binding stability of the chemical components with α-Glu protein. The seven compounds screened from *PC* showed a higher affinity. It was speculated that the conjugated structure of the candidate components affected additional interactions. Table 1 showed in detail the binding energies, number of hydrogen bonds, and binding pockets of α-Glu with acarbose and the active compounds. The properties revealed that the seven selected components might also be good inhibitors, as well as further verify the practicality of the method.

## 4 Conclusion

A fluorometric nanocomposite sensor was successfully designed based on R-CDs and CoOOH NSs for detecting α-Glu. A simple and automatic separation and collection system was established for screening active ingredients from *PC* extracts in combination with the sensor. The fluorescent sensor had been proved to be easy to operate, low-cost, and sensitive. It was made up of FRET between R-CDs (λem = 625 nm) and CoOOH NSs, particularly demonstrating strong resistance to matrix interference. Upon separation and preparation of *PC* extracts with HPLC-DAD-FC, the inhibiting efficiency of the seven potential active ingredients with α-Glu inhibitorary activity was monitored. They were also recognized as α-Glu inhibitors through experimental and computer-assisted evaluation. Thus, this work not only holded significance for detecting enzyme activities and screening α-Glu inhibitors, but also was greatly promising for exploring new antidiabetic drugs from natural products.

## CRediT authorship contribution statement

**Huihui Sun:** Writing – original draft, Validation, Methodology, Investigation, Data curation, Conceptualization. **Chuanyuan Gao:** Software, Resources, Conceptualization. **Yumin Yang:** Software, Resources. **Changqing Liu:** Resources, Conceptualization. **Han Qin:** Investigation. **Mengyuan Tan:** Investigation. **Jin Li:** Writing – review & editing, Investigation. **Xiaoxia Li:** Conceptualization. **Kunze Du:** Writing – review & editing, Funding acquisition, Conceptualization. **Yanxu Chang:** Supervision, Project administration, Funding acquisition, Conceptualization.

## Declaration of competing interest

The authors declare that they have no known competing financial interests or personal relationships that could have appeared to influence the work reported in this paper.

## Data Availability

Data will be made available on request.
