# Supplementary material for: Red-emissive carbon dot-cobalt oxyhydroxide nanosystem: A turn-on sensor for α-Glucosidase activity and inhibitor identification
Source: Mater Today Bio. 2025 Jun 23;33:102018. doi: 10.1016/j.mtbio.2025.102018 (PMC12264606; doi:10.1016/j.mtbio.2025.102018)
Supplement: Multimedia component 1 [file mmc1.docx]

**Supplementary Material**

**Red-emissive carbon dot-cobalt oxyhydroxide nanosystem: A turn-on sensor for α-Glucosidase activity and inhibitor identification**

Huihui Sun ^a,b,1^, Chuanyuan Gao ^a,b,1^, Yumin Yang ^c^, Qingchang Liu ^a,b^, Han Qin ^a,b^,Mengyuan Tan ^a,b^, Jin Li ^a,b^, Xiaoxia Li ^a,c^, Kunze Du ^a,b,*^, Yanxu Chang ^a,b,**^

*^a^ State Key Laboratory of Chinese Medicine Modernization, Tianjin University of Traditional Chinese Medicine, Tianjin, 301617, China*

*^b^ Tianjin Key Laboratory of Phytochemistry and Pharmaceutical Analysis, Tianjin University of Traditional Chinese Medicine, Tianjin, 301617, China*

*^c^* *School of Chinese Materia Medica, Tianjin University of Traditional Chinese Medicine, Tianjin, 300193, China*

Corresponding Author

^*^ Kunze Du, E-mail: dkztcm@tjutcm.com

^**^ Yanxu Chang, E-mail: [tcmcyx@tjutcm.edu.cn](mailto:tcmcyx@tjutcm.edu.cn)

^1^ The authors contributed equally to the first author


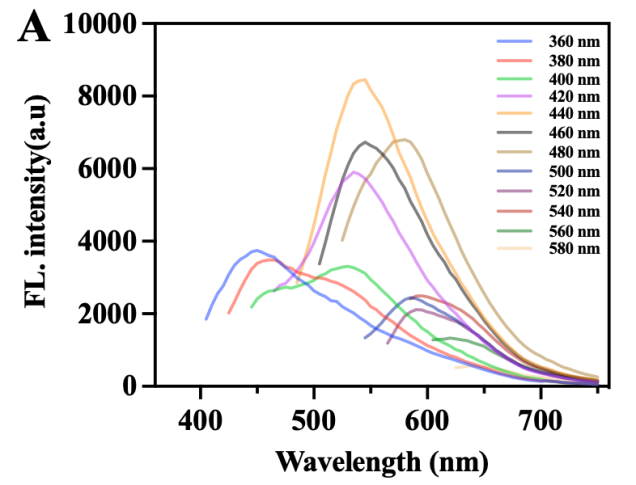

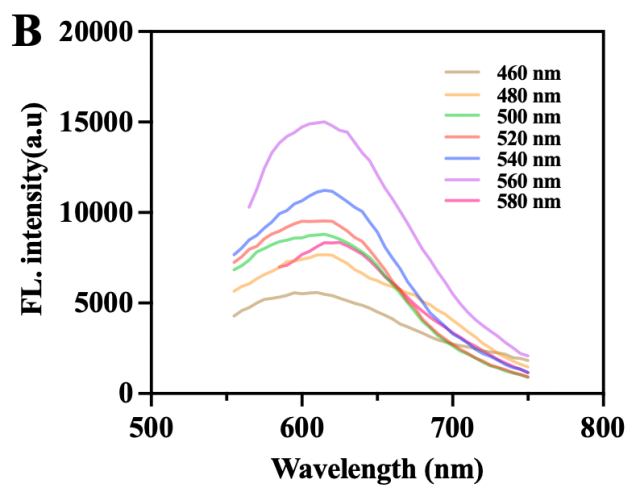


**Fig. S1** Fluorescence emission spectra of unpurified R-CDs (A) and purified R-CDs (B).


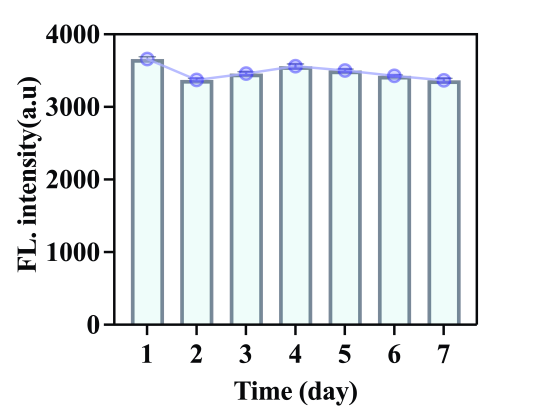


**Fig. S2** Fluorescence intensity of R-CDs during seven days.


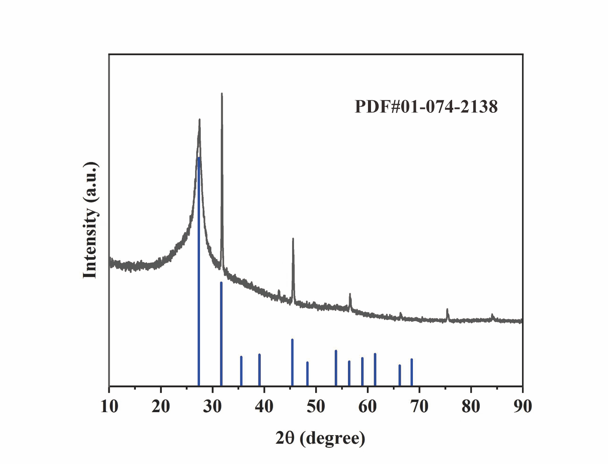


**Fig. S3** XRD spectra of R-CDs.


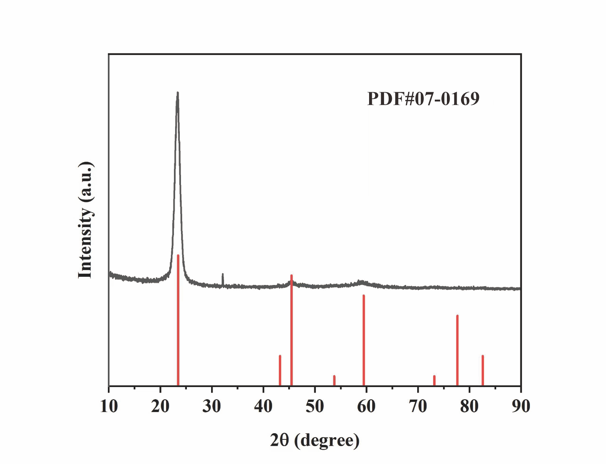


**Fig. S4** XRD spectra of CoOOH NSs.

R-CDs (50 μL) were mixed with different volumes of CoOOH NSs (0, 5, 10, 15, 20, 25 μL), and then ultrapure water was added to 100 μL. The mixture was incubated at 37°C for 20 minutes and then centrifuged at 8000 rpm for 8 minutes. The fluorescence intensity of the supernatant was measured.

The loading rate (%) = (F-F_1_)/(F-F_0_), F was the fluorescence intensity of the R-CDs without CoOOH NSs; F_1_ was the fluorescence intensity of the supernatant in the CoOOH NSs added groups; F_0_ was the fluorescence intensity of the supernatant without CoOOH NSs and R-CDs.


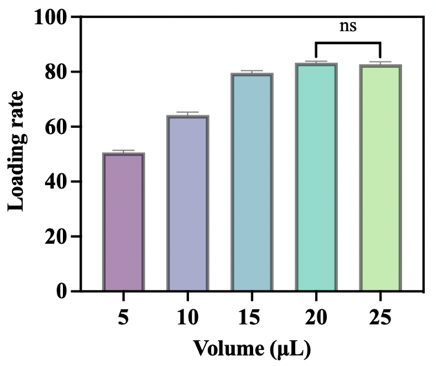


**Fig. S5** The loading rate of R-CDs on CoOOH NSs (N=3, Error bars are the standard error of the mean).


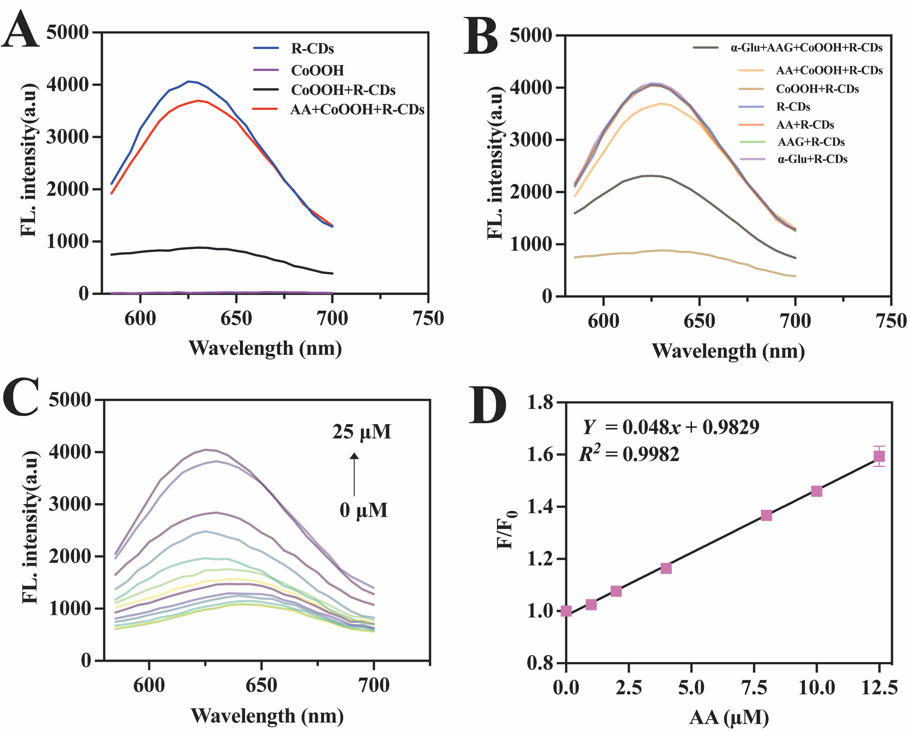


***Y* = 0.0480*x*+0.9829**

***R*^2^ = 0.9982**

**Fig. S6** (A) Fluorescence emission spectra of R-CDs, CoOOH NSs, and R-CDs@CoOOH NCs. (B) Fluorescence emission spectra of R-CDs with various substances. (AA:12.5 μM, AAG: 4 mM, α-Glu: 10 U mL^-1^) (C) Fluorescence spectra of R-CDs@CoOOH NCs at different AA activity (0 - 25 μM). (D) The linear relationship between the fluorescence intensity of R-CDs@CoOOH NCs and AA (0-12.5 μM).


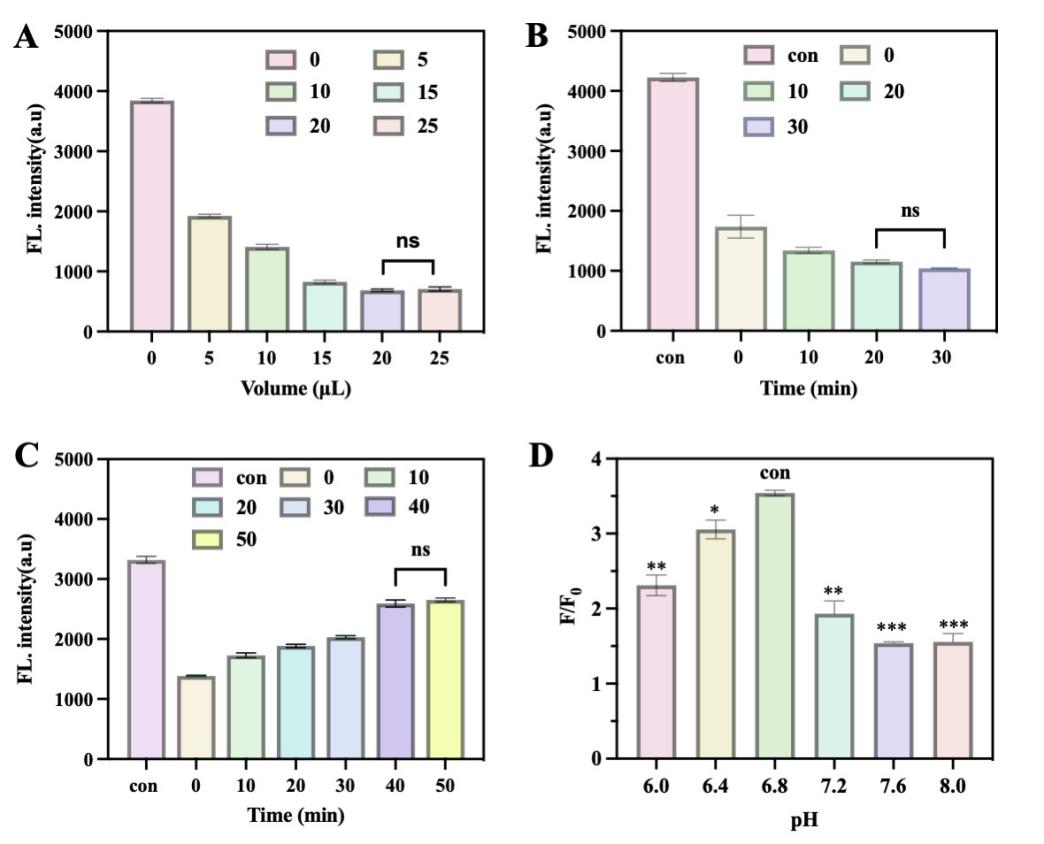


**Fig. S7** Schematic diagram of optimized reaction conditions. (A) Optimization of the reaction ratio between R-CDs and CoOOH NSs; (B) Optimization of the reaction time between R-CDs and CoOOH NSs; (C) Optimization of the reaction time between AA (25μM) and R-CDs@CoOOH NCs; (D) Optimization of pH for the α-Glu reaction. (N=3; * *P*<0.05, ** *P*<0.01, *** *P*<0.001)


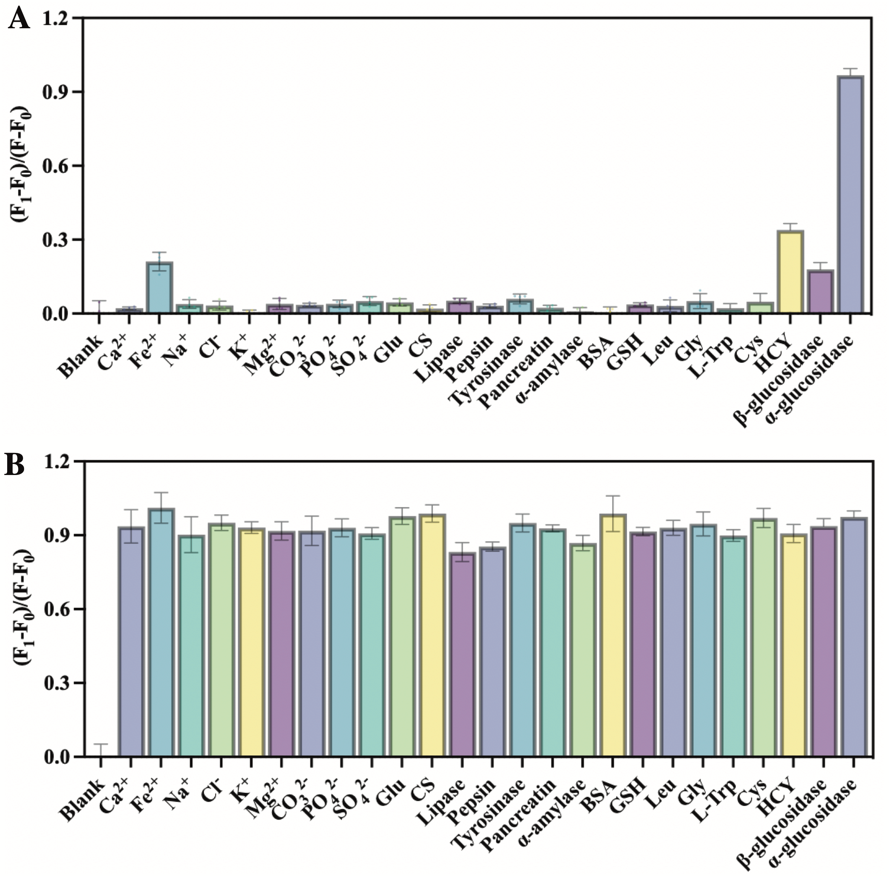


**Fig. S8** Selectivity (A) and anti-interference (B) analysis for α-Glu activities in fluorometric mode. The concentration of inorganic ions, biomolecules, and amino acids was 1 mM and the concentration of enzymes was 10 U mL^-1^.

**
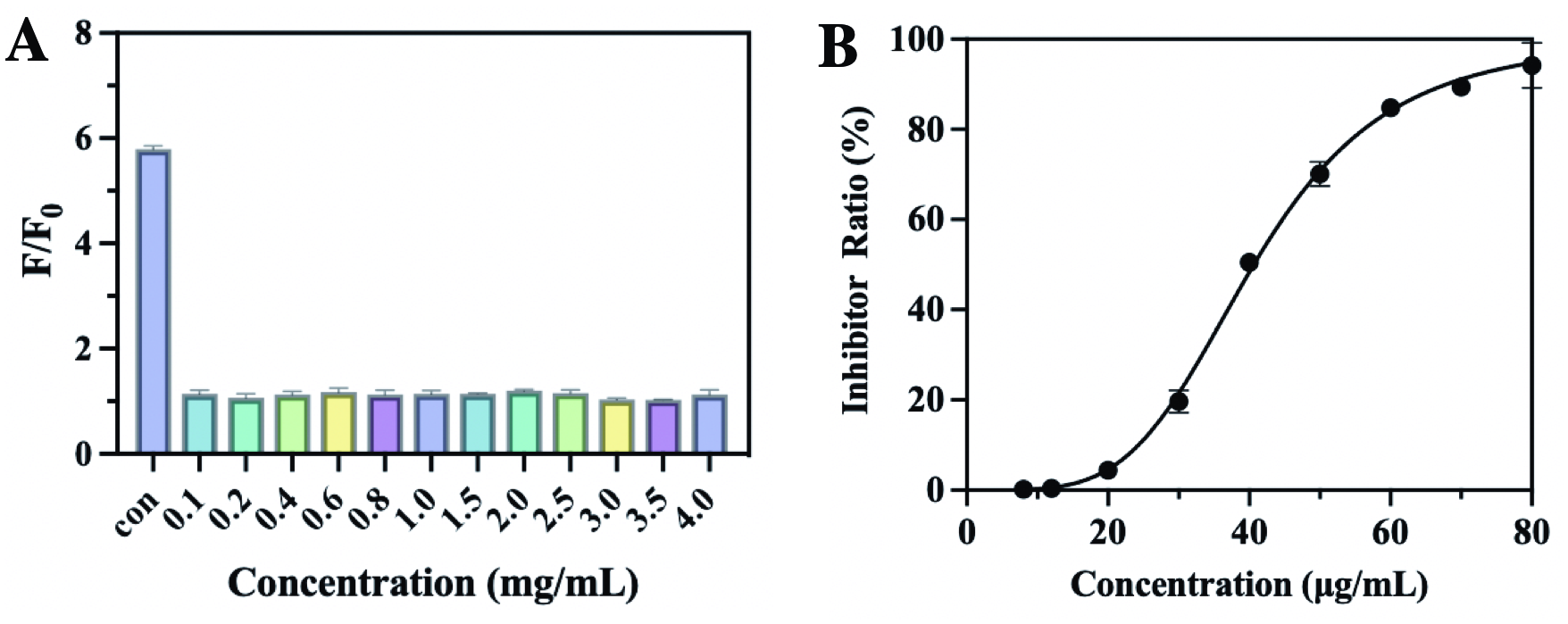
**

**Fig. S9** (A) The reducing effect of different concentrations of *PC* extracts (Con: the fluorescence intensity in the presence of enzymes and substrates); (B) Screening the inhibition curve of *PC* extracts using traditional colorimetric method (IC_50_=40.63 μg mL^-1^, *R*^2^=0.9969).

**Fig. S10** The chromatograms of negative ions.

**Fig. S11** The chromatograms of positive ions.





**Fig. S12** HPLC chromatogram of *PC* extracts and seven components (1) Polydatin, (2) (-)-Epicatechin gallate, (3) Emodin-1-*O*-D-glucoside, (4) Resveratrol, (5) Torachrysone-8-*O*-D-glucoside, (6) Emodin-8-*O*-D-glucoside, (7) Emodin.


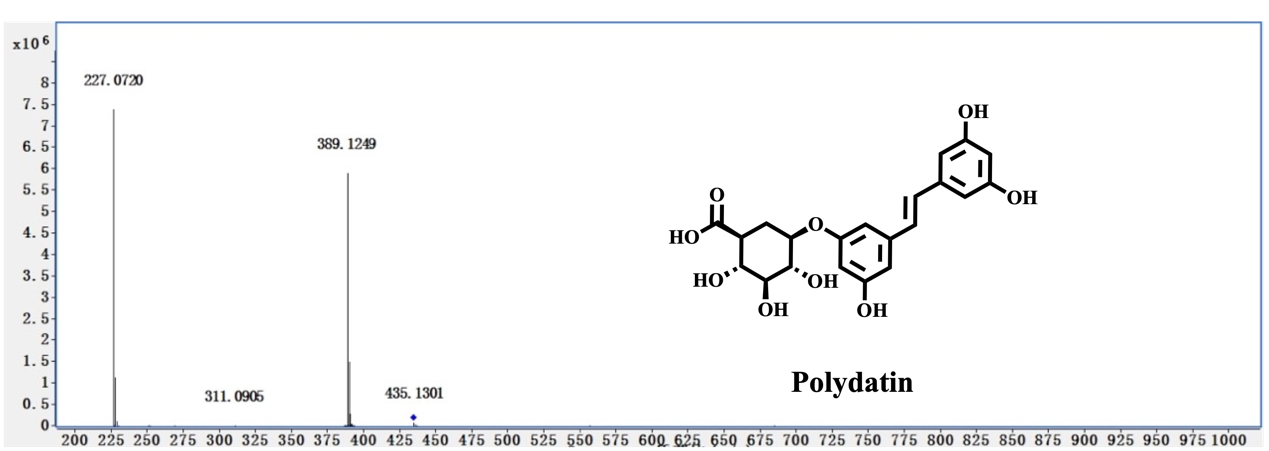


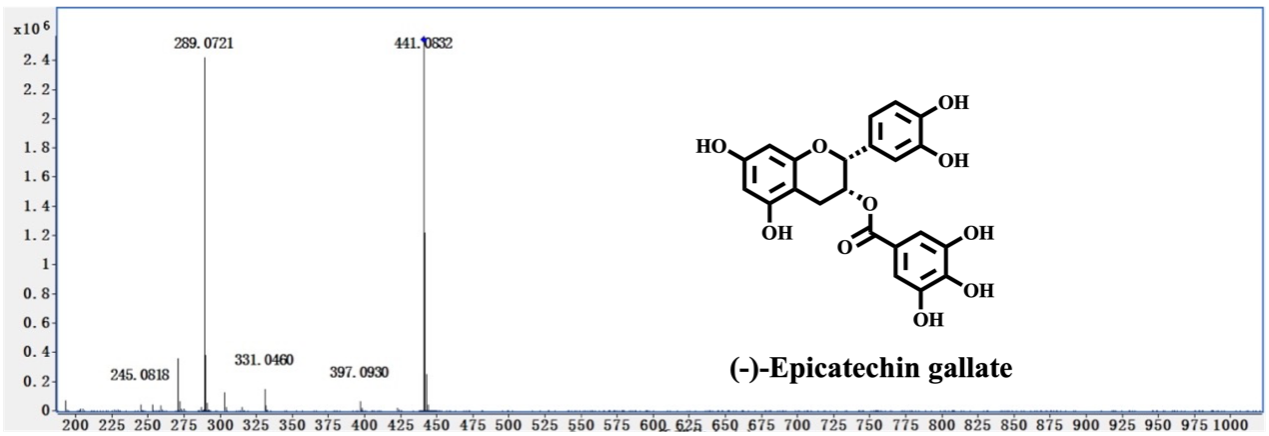


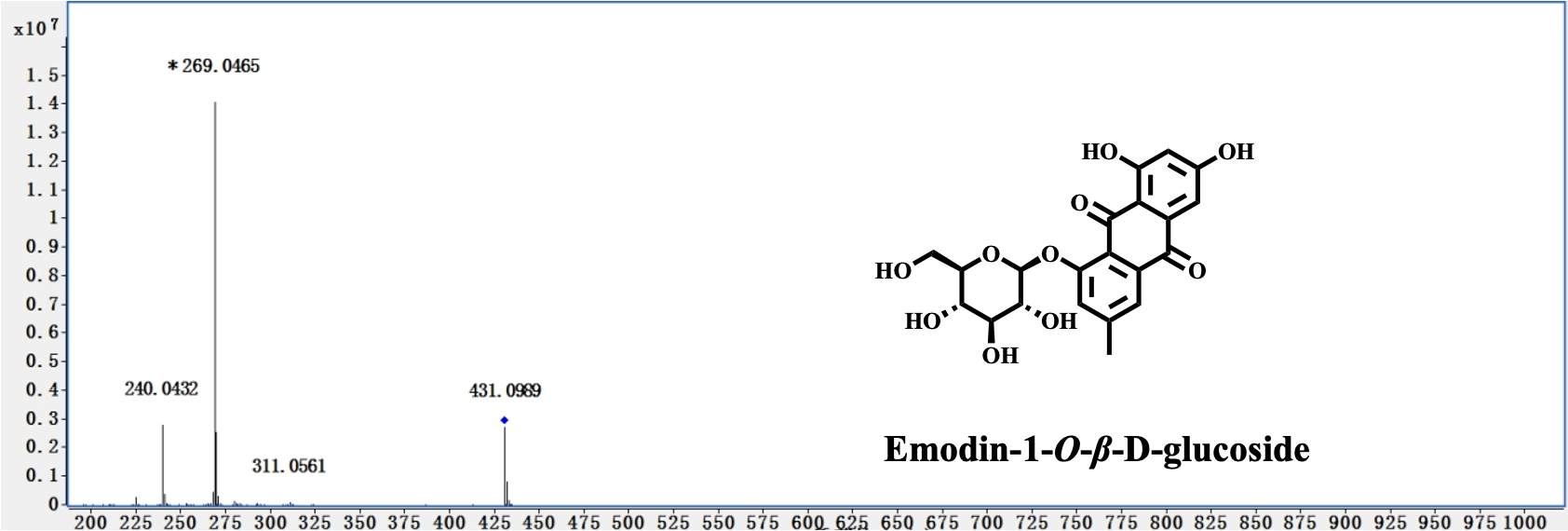


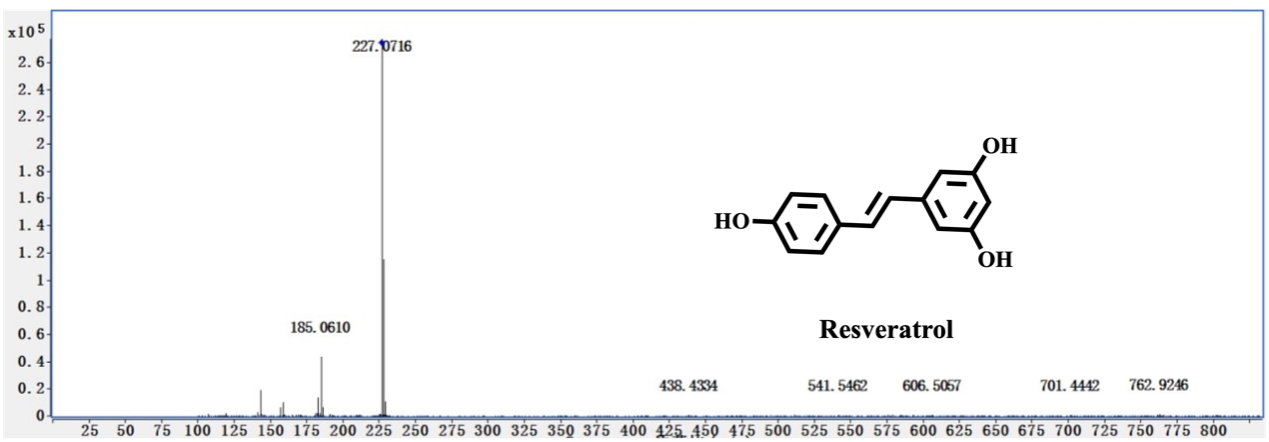


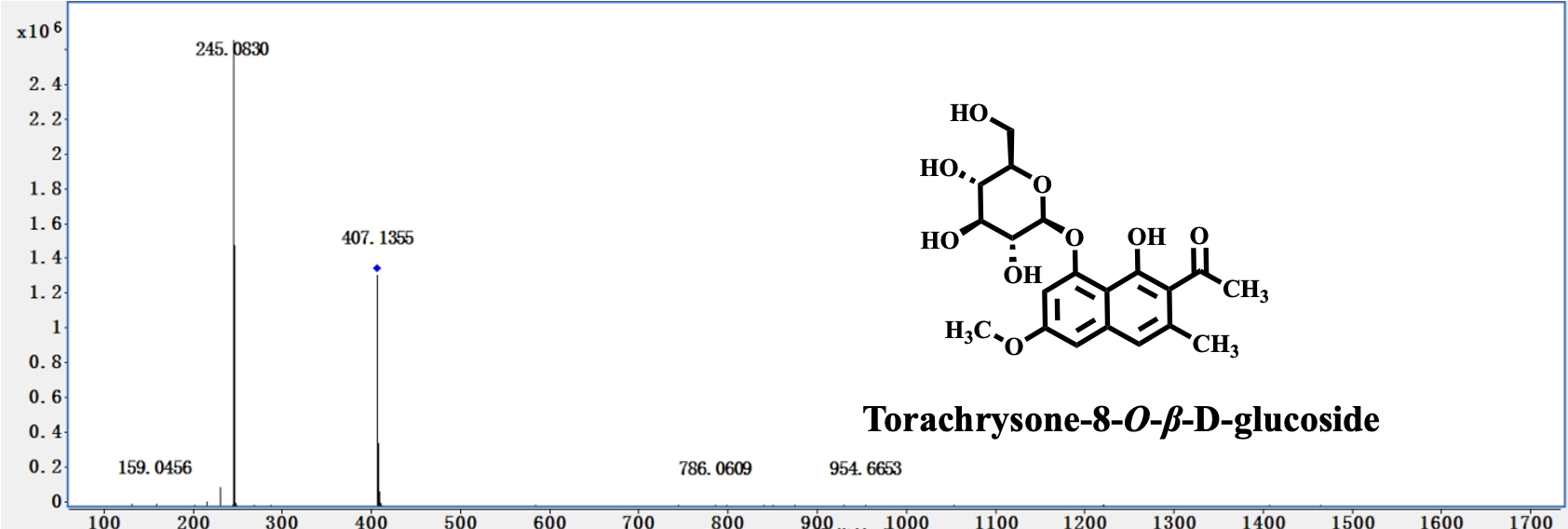


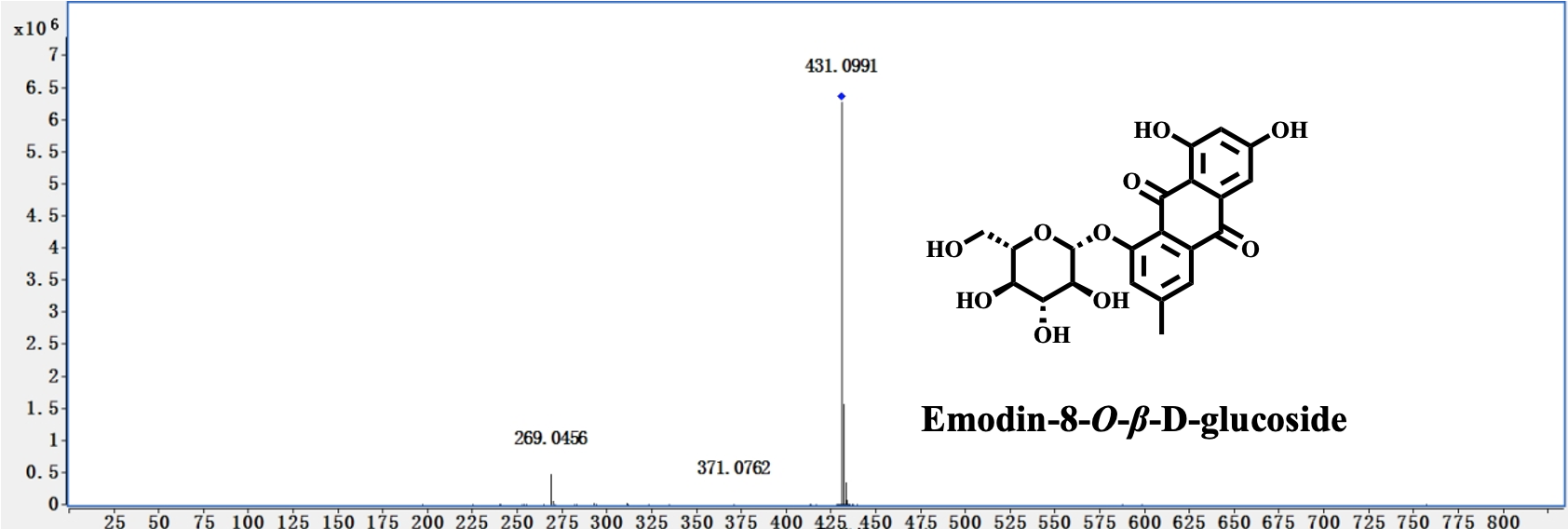


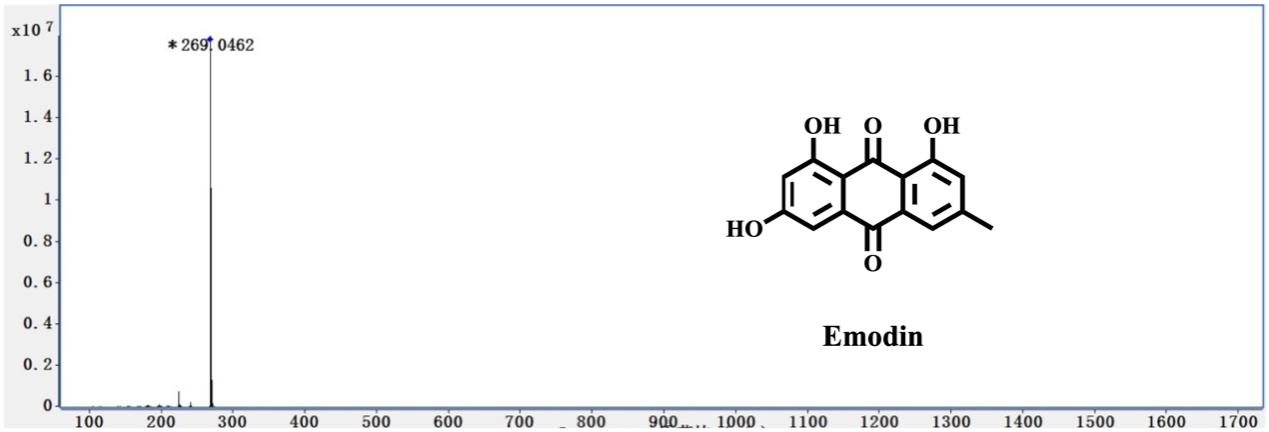


**Fig. S13** Mass spectrometry secondary information and chemical structure of seven active ingredients.

**Table S1** Comparison with previously studied assays

| Materials | Mode | Linear range | Linear regression equation | LOD | Reference |
| --- | --- | --- | --- | --- | --- |
| CoOOH nanozymes | Colorimetry | 0.01 - 0.4 U/mL | Y = 0.087x + 0.4059 (R^2^ = 0.9920) | 0.0048 U/mL | [1] |
| Single-atom Pt | Colorimetry | - 1. - 8 U/mL | Y = 0.111x+0.0024 (R^2^ = 0.9960) | 0.0038 U/mL | [2] |
| AuNPs | Colorimetry | 0.05 - 1.1 U/mL | Y = –1.112 x + 1.358 (R^2^ = 0.9890) | 0.004 U/mL | [3] |
| Nitrogen-doped carbon dots | Fluorometry | 0.2 - 1 U/mL | Y = 76.07x + 33.52 (R^2^ = 0.9948) | 0.01 U/mL | [4] |
| AgInZnS QDs | Fluorometry | 0.01 - 0.16 U/mL | Y = 12.325x + 1.046 (R2 = 0.9904) | 0.0073 U/mL | [5] |
| Carbon dots | Fluorometry | 0 - 5.5 U/mL | R^2^ = 0.9930 | 0.02 U/mL | [6] |
| Ag nanoclusters/  MnO2 nanosheet | Ratiometric fluorescence | 0.2 - 8.0 U/mL | Y = 0.1619x + 1.6311 (R^2^ = 0.9974) | 0.03 U/mL | [7] |
| WS2 QDs/CoOOH nanosheet | Ratiometric fluorescence | 0.05 - 10 U/mL | Y = − 0.0564x + 0.7967 (R^2^ = 0.9911) | 0.009 U/mL | [8] |
| R-CDs@CoOOH NCs | Fluorometry | 0.01 - 15 U/mL | Y = 0.2706x + 0.9745 (R^2^ = 0.9926) | 0.0037 U/mL | in this work |

**Table S2** Identification of 85 chemical constituents of *PC* extracts by UPLC-Q-TOF/MSMS

| Peak | T_R_ | m/z[M-H]^-^ | m/z[M+H]^-^ | Formula | Error(ppm) | MS^2^ | Identification |
| --- | --- | --- | --- | --- | --- | --- | --- |
| 1 | 1.1760 | 377.0863 | - | C_18_H_17_O_9_ | 1.09 | 341.1085, 179.0558, 161.0456 | Unkown carbohydrate |
| 2 | 1.5300 | 133.0145 | - | C_4_H_6_O_5_ | -1.9 | 115.0040, 107.0366 | Malic acid |
| 3 | 1.6700 | 191.0203 | - | C_6_H_8_O_7_ | 2.99 | 111.0087 | Citric Acid |
| 4 | 2.0950 | 331.0678 | - | C_13_H_16_O_10_ | -2.2 | 271.0464, 211.0252, 169.-246, 125.0246 | 6-O-Galloylglucose |
| 5 | 2.4160 | 169.0146 | - | C_7_H_6_O_5_ | -2.68 | 125.0247 | Gallic acid |
| 6 | 3.4140 | 315.0727 | - | C_13_H_14_O_9_ | - | 254.8570, 152.0118, 108.0219 | Salicylic acid *O*-*β*-D-glucoside |
| 7 | 3.5120 | 323.1352 | - | C_13_H_23_O_9_ | -4.78 | 254.8556, 101.0245 | Unkown |
| 8 | 4.5030 | 153.0196 | - | C_7_H_6_O_4_ | -1.75 | 109.0299 | Protocatechuic acid |
| 9 | 4.7160 | 357.0837 | - | - | - | 283.8792, 182.0223, 151.0404, 107.0504 | Unkown |
| 10 | 5.4230 | 197.8080 | - | - | - | - | Unkown |
| 11 | 5.5650 | 451.1248 | - | C_21_H_24_O_11_ | -0.48 | 289.0714, 245.0818, 125.0244 | (+)-catechin 3'-*O*-*β*-D-glucopyranoside |
| 12 | 5.9190 | 577.1364 | - | C_30_H_26_O_12_ | -2.71 | 479.0839, 425.0881, 368.9599, 289.0727, 125.0247 | Procyanidin B1 |
| 13 | 6.2730 | 353.0883 | - | C_16_H_18_O_9_ | -2.25 | 191.0563, 127.0402 | Chlorogenic acid |
| 14 | 6.4150 | 577.1364 | - | C_30_H_26_O_12_ | -2.71 | 479.0839, 425.0881, 368.9599, 289.0727, 125.0247 | Procyanidin B2 |
| 15 | 6.5570 | 353.0876 | - | C_16_H_17_O_9_ | -1.68 | 191.0563 | Chlorogenic acid |
| 16 | 6.6280 | 289.0725 | - | C_15_H_14_O_6_ | -2.55 | 245.0824, 125.0245 | (+)-catechin |
| 17 | 6.9820 | 329.0882 | - | C_14_H_18_O_9_ | -1.19 | 167.0353, 123.0454 | Vanillic acid 4-*β*-D-glucoside |
| 18 | 7.2670 | - | 417.1177 | C_21_H_20_O_9_ | 0.74 | 255.0641 | Chrysophanol-8-*O*-glucoside |
| 19 | 7.4090 | - | 419.1333 | C_21_H_22_O_9_ | 0.86 | 255.0640, 201.0090 | Polygonimitin B |
| 20 | 7.4780 | 577.1364 | - | C_30_H_26_O_12_ | -2.71 | 479.0839, 425.0881, 368.9599, 289.0727, 125.0247 | Procyanidin B4 |
| 21 | 7.7400 | 299.0563 | - | C_16_H_12_O_6_ | -0.63 | - | Fallacinol |
| 22 | 7.9740 | 469.0819 | - | C_16_H_21_O_16_ | 3.42 | 389.1239, 306.0199, 227.0718, 138.9709 | Unkown |
| 23 | 8.0450 | 289.0723 | - | C_15_H_14_O_6_ | -1.86 | 245.0824, 125.0245 | Epicatechin |
| 24 | 8.3280 | 729.1478 | - | C_37_H_30_O_16_ | -2.32 | 635.0915, 407.0777, 289.0725, 205.0513, 125.0246 | Procyanidin-B1-7-3-*O*-gallate |
| 25 | 8.5410 | 405.1197 | - | C_20_H_22_O_9_ | -1.47 | 367.1038, 243.0666 | Piceatannol-3'-*O*-*β*-D-glucopyranoside |
| 26 | 8.6820 | 435.1302[(M+COOH)-] | - | C_20_H_22_O_8_ | -1.22 | 389.1252, 227.0721 | Resveratroloside |
| 27 | 8.7530 | 435.1302[(M+COOH)-] | - | C_20_H_22_O_8_ | -1.22 | 389.1252, 227.0721 | (+)-Polydatin |
| 28 | 9.0360 | 729.1488 | - | C_37_H_30_O_16_ | -3.69 | 577.1359, 451.1039, 407.0779, 289.0723, 169.0146, 125.0247 | Procyanidin-B2-7-3-*O*-gallate |
| 29 | 9.5320 | 469.0825 | - | C_16_H_21_O_16_ | 2.15 | 389.1264, 299.0934, 241.0030, 138.9710 | Unkown |
| 30 | 9.8190 | 447.0929 | - | C_21_H_20_O_11_ | 0.86 | 285.0401 | Luteolin-7-*O*-*β*-D-glucoside |
| 31 | 10.0280 | 425.1023[(M+Cl)-] | - | C_20_H_22_O_8_ | -3.37 | 389.1228, 227.0719 | Polydatin |
| 32 | 10.3820 | 441.0838 | - | C_22_H_18_O_10_ | -2.45 | 397.0931, 331.0463, 289.0725, 169.0148, 125.0246 | Catechin-3-*O*-gallate |
| 33 | 10.5240 | 441.0827 | - | C_22_H_18_O_10_ | -1.77 | 397.0931, 331.0463, 289.0725, 169.0148, 125.0246 | (–)-Epicatechin-3-*O*-gallate |
| 34 | 10.8780 | 205.0512 | - | C_11_H_10_O_4_ | -2.77 | 161.0247, 106.0426 | 7-Hydroxy-4-methoxy-5-methyl coumarin |
| 35 | 10.9490 | 541.1367 | - | C_27_H_26_O_12_ | -2.86 | 475.0891, 389.1254, 313.0569, 227.0717, 169.0146 | Resveratrol-4'-*O*-(6"-galloyl)glucoside |
| 36 | 11.3030 | 541.1365 | - | C_27_H_26_O_12_ | -1.76 | 313.0571, 227.0718, 169.0146 | Resveratrol-4-*O*-  (2'′-galloyl)-glucopyranoside |
| 37 | 11.6570 | 541.1369 | - | C_27_H_26_O_12_ | -3.23 | 313.0572, 227.0719, 169.0147 | 3,5,4'-Trihydroxystilbene-4-*O*-(6''-galloyl)-glucopyranoside |
| 38 | 11.7980 | 487.0931 | - | C_16_H_25_O_17_ | 1.99 | 407.1344, 245.0827, 183.0298, 125.0251, 287.0931 | Undown |
| 39 | 12.2240 | - | 417.1172 | C_21_H_20_O_9_ | 1.94 | 255.0641 | Chrysophanol-8-*O*-glucoside |
| 40 | 12.3650 | 541.1367 | - | C_27_H_26_O_12_ | -2.86 | 313.0572, 227.0719, 169.0147 | 3,5,4'-Trihydroxystilbene-4-*O*-(2''-galloyl)-glucopyranoside |
| 41 | 12.5780 | 431.1002 | - | C_21_H_20_O_10_ | -4.24 | 311.0568, 269.0461, 240.0434 | Aloe-emodin-1-*O*-glucoside |
| 42 | 13.0020 | 431.1002 | - | C_21_H_20_O_10_ | -4.24 | 311.0568, 269.0461, 240.0434 | Aloe-emodin-8-*O*-glucoside |
| 43 | 13.1420 | 259.0612 | - | C_14_H_12_O_5_ | -0.01 |  | 2-Methoxy-6-acetyl-7-methyljuglone |
| 44 | 13.1440 | 511.0563 | - | - | - | 269.0459, 241.0028 | Anthraglycoside B sulfate |
| 45 | 13.2860 | 269.0464 | - | C_15_H_10_O_15_ | -3.17 | 225.0566 | Genistein |
| 46 | 13.4980 | 227.0720 | - | C_14_H_12_O_3_ | -2.78 | 227.0716 | Resveratrol |
| 47 | 13.7110 | 583.1104 | - | C_28_H_24_O_14_ | -1.83 | 431.0986, 269.0461, 241.0358, 169.0146 | Emodin-1-*O*-(galloyl)-glucoside |
| 48 | 13.9230 | 583.1104 | - | C_28_H_24_O_14_ | -1.83 | 431.0986, 269.0461, 241.0358, 169.0146 | Emodin-8-*O*-(galloyl)-glucoside |
| 49 | 14.0672 | 463.0883 | - | C_21_H_20_O_12_ | -3.88 | 343.0458, 300.0278, 271.0248, 178.9987, 151.0038 | Isoquercitrin |
| 50 | 14.2770 | 205.0508 | - | C_11_H_9_O_4_ | -0.81 | 190.0273, 161.0247, 106.0426 | Unkown |
| 51 | 14.6330 | 407.1362 | - | C_20_H_24_O_9_ | -3.55 | 287.0923, 245.0828, 159.0455 | Torachrysone-8-*O*-D-glucoside |
| 52 | 14.9160 | 431.0000 | - | C_21_H_20_O_10_ | -4.24 | 311.0568, 269.0461, 225.0564 | Emodin-1-*O*-*β*-D-glucoside |
| 53 | 15.2000 | 431.1004 | - | C_21_H_20_O_10_ | -2.38 | 269.0461, 241.0493, 225.0540 | Emodin-8-*O*-*β*-D-glucoside |
| 54 | 15.2700 | 511.0562 | - | - | - | 511.0566, 431.0990, 241.0028, 269.0462 | Emodin-*O*-glucoside sulfate |
| 55 | 15.4120 | 583.1103 | - | - | - | 431.0986, 269.0461, 241.0358, 169.0146 | Emodin-8-*O*-(galloyl)-glucoside |
| 56 | 15.6240 | 525.0715 | - | - | - | 431.0923, 283.0623, 241.0029 | Physcion-8-*O*-glucoside sulfate |
| 57 | 15.8380 | 517.1003 | - | C_24_H_22_O_13_ | -2.97 | 473.1098, 431.0989, 311.0565, 269.0463, 240.0434 | Emodin-8-*O*-(6'-methylmalonyl)-glucopyranoside |
| 58 | 16.0490 | 449.1459 | - | C_22_H_26_O_10_ | -1.29 | 389.1248, 287.0944, 245.0825, 159.0463 | Torachryson-8-*O*-(6'-acetyl)-glucoside |
| 59 | 16.2620 | 283.0619 | - | C_16_H_12_O_5_ | -2.48 | 240.0432, 154.8989 | Physcion |
| 60 | 16.4740 | 779.2202 | - | - | - | 633.1832, 487.1459, 289.0724 | Hydropiperoside |
| 61 | 16.6870 | 283.0619 | - | C_16_H_12_O_5_ | -2.48 | 240.0432, 154.8989 | Questin |
| 62 | 16.8280 | 481.0914 | - | C_28_H_18_O_8_ | 3.09 | 431.0979, 366.8098, 283.0616, 240.0434, 125.0248 | Unkown |
| 63 | 17.1830 | 245.2600 | - | C_14_H_14_O_4_ | -4.76 | 215.0360, 159.0457 | Torachrysone |
| 64 | 17.7490 | 301.0351 | - | C_15_H_10_O_7_ | 0.59 | 245.0454, 178.9988, 151.0035, 121.0294 | Quercetin |
| 65 | 17.9290 | 285.0407 | - | C_15_H_10_O_6_ | -0.83 | 133.0295 | Luteolin |
| 66 | 18.2460 | 245.0826 | - | - | - | 215.0356, 187.0406, 159.0456, 131.0506 | Unkown |
| 67 | 19.5900 | 671.1783 | - | - | - | 509.1252, 416.1117, 254.0592 | Emodin dianthrone hexose |
| 68 | 19.8740 | 671.1783 | - | - | - | 509.1252, 416.1117, 254.0592 | Emodin dianthrone hexose |
| 69 | 19.9450 | - | 261.0769 | C_14_H_12_O_5_ | -4.41 | 215.0709, 159.0446 | 2-Methoxy-6-acetyl-7-methyljuglone |
| 70 | 20.1570 | 162.8394 | - | - | - | 100.9571 | Unkown |
| 71 | 20.3690 | 329.0674 | - | C_17_H_14_O_7_ | -2.19 | 299.0201, 271.0256, 243.0311, 252.9177 | 3',5,7-Trihydroxy-3,4'-dimethoxy |
| 72 | 20.5820 | 349.0034 | - | - | - | 269.0464 | Emodin sulfate |
| 73 | 20.6520 | 349.0034 | - | - | - | 269.0465, 225.0564, 105.0348 | Emodin sulfate |
| 74 | 20.9360 | 269.0459 | - | C_15_H_10_O_5_ | -1.31 | 225.0561, 197.0611 | Aloe-emodin |
| 75 | 21.2900 | 671.1783 | - | - | - | 509.1252, 416.1117, 254.0592 | Emodin dianthrone hexose |
| 76 | 21.6460 | 685.1942 | - | - | - | 269.0462, 225.0564, 170.8830, 101.8677 | Emodin sulfate |
| 77 | 21.9290 | 671.1783 | - | - | - | 509.1252, 416.1117, 254.0592 | Emodin dianthrone hexose |
| 78 | 23.1330 | 685.1939 | - | - | - | 568.1918, 485.6935, 416.1120, 254.0588, 226.0637 | Methyl derivative of emodin dianthrone hexose |
| 79 | 23.7800 | 685.1939 | - | - | - | 568.1918, 485.6935, 416.1120, 254.0588, 226.0637 | Methyl derivative of emodin dianthrone hexose |
| 80 | 24.1240 | 311.0569 | - | - | - | 268.0385, 240.0430, 180.9115 | Unkown |
| 81 | 24.8510 | 593.2744 | - | - | - | 530.0649, 413.2094, 315.0487, 277.2183, 241.0126, 152.9959 | Unkown |
| 82 | 25.0640 | 269.0464 | - | C_15_H_10_O_5_ | -3.17 | 225.0563, 197.0612, 105.0350 | Emodin |
| 83 | 27.0460 | 509.1249 | - | C_30_H_22_O_8_ | -1.39 | 491.1122, 254.0589 | (+)-Emodin bianthrone |
| 84 | 27.8250 | 509.1249 | - | C_30_H_22_O_8_ | -1.39 | 491.1122, 254.0589 | (-)-Emodin bianthrone |
| 85 | 28.1080 | 489.3443 | - | - | - | 423.3113, 369.3006, 236.8579, 164.9281, 164.9281, 101.0244 | Unkown |

**Reference**

[1] D. Ma, J. Ge, A. Wang, J. Li, H. Yang, W. Zhai, R. Cai, Ultrasensitive determination of α-glucosidase activity using CoOOH nanozymes and its application to inhibitor screening, J. Mat. Chem. B 11(12) (2023) 2727-2732.

[2] G. Kang, W. Liu, F. Liu, Z. Li, X. Dong, C. Chen, Y. Lu, Single-atom Pt catalysts as oxidase mimic for p-benzoquinone and α-glucosidase activity detection, Chemical Engineering Journal/Chem. Eng. J. 449 (2022) 137855.

[3] J. Zhang, Y. Liu, J. Lv, G. Li, A colorimetric method for α-glucosidase activity assay and its inhibitor screening based on aggregation of gold nanoparticles induced by specific recognition between phenylenediboronic acid and 4-aminophenyl-α-d-glucopyranoside, Nano Res. 8(3) (2014) 920-930.

[4] W. Kong, D. Wu, L. Xia, X. Chen, G. Li, N. Qiu, G. Chen, Z. Sun, J. You, Y. Wu, Carbon dots for fluorescent detection of α-glucosidase activity using enzyme activated inner filter effect and its application to anti-diabetic drug discovery, Anal. Chim. Acta 973 (2017) 91-99.

[5] J. Zhang, J. Liu, M. Wang, G. Wang, X. Su, A fluorometric assay for α-glucosidase activity based on quaternary AgInZnS QDs, Microchim. Acta 188(7) (2021) 227.

[6] J. Liu, F. Wu, C. Liu, H. Bao, T. Fu, “Turn-on” fluorometric probe for α-glucosidase activity using red fluorescent carbon dots and 3,3′,5,5′-tetramethylbenzidine, Microchim. Acta 187(9) (2020) 498.

[7] M. Shi, Y. Cen, G. Xu, F. Wei, X. Xu, X. Cheng, Y. Chai, M. Sohail, Q. Hu, Ratiometric fluorescence monitoring of α-glucosidase activity based on oxidase-like property of MnO2 nanosheet and its application for inhibitor screening, Anal. Chim. Acta 1077 (2019) 225-231.

[8] Z. Zhai, W. Wang, Z. Chai, Y. Yuan, Q. Zhu, J. Ge, Z. Li, A ratiometric fluorescence platform based on WS2 QDs/CoOOH nanosheet system for α-glucosidase activity detection, Spectroc. Acta Pt. A- Molec. Biomolec. Spectr. 310(5) (2024) 123959.
